# *Datura stramonium* Flowers as a Potential Natural Resource of Bioactive Molecules: Identification of Anti-Inflammatory Agents and Molecular Docking Analysis

**DOI:** 10.3390/molecules28135195

**Published:** 2023-07-04

**Authors:** Bilel Damergi, Rym Essid, Nadia Fares, Nadine Khadraoui, Lucía Ageitos, Ameni Ben Alaya, Dorra Gharbi, Islem Abid, Monerah Rashed Alothman, Ferid Limam, Jaime Rodríguez, Carlos Jiménez, Olfa Tabbene

**Affiliations:** 1 Laboratory of Bioactive Substances, Biotechnology Center of Borj Cedria, BP-901, Hammam-Lif 2050, Tunisia; 2 Faculty of Sciences of Tunis, University of Tunis El Manar, Tunis 2092, Tunisia; 3 Centro Interdisciplinar de Química e Bioloxía (CICA) and Departamento de Química, Facultade de Ciencias, Universidade da Coruña, 15071 Coruña, Spain; 4 Botany and Microbiology Department, Science College, King Saud University, Riyadh 11451, Saudi Arabia

**Keywords:** anti-inflammatory, bioactive molecules, *Datura stramonium*, molecular docking, HR-ESI-MS, NMR

## Abstract

The present study investigated the antioxidant, antibacterial, antiviral and anti-inflammatory activities of different aerial parts (flowers, leaves and seeds) of *Datura stramonium*. The plant material was extracted with 80% methanol for about 24 h. The sensitivity to microorganisms analysis was performed by the microdilution technique. Antioxidant tests were performed by scavenging the DPPH and ABTS radicals, and by FRAP assay. Anti-inflammatory activity was evaluated through the inhibition of nitric oxide production in activated macrophage RAW 264.7 cells. Cell viability was assessed with an MTT assay. Results show that the flower extract revealed a powerful antimicrobial capacity against Gram-positive bacteria and strong antioxidant and anti-inflammatory activities. No significant cytotoxicity to activated macrophages was recorded. High resolution electrospray ionization mass spectrometry and nuclear magnetic resonance analysis identified two molecules with important anti-inflammatory effects: 12α-hydroxydaturametelin B and daturametelin B. Molecular docking analysis with both pro-inflammatory agents tumor necrosis factor alpha and interleukin-6 revealed that both compounds showed good binding features with the selected target proteins. Our results suggest that *D. stramonium* flower is a promising source of compounds with potential antioxidant, antibacterial, and anti-inflammatory activities. Isolated withanolide steroidal lactones from *D. stramonium* flower extract with promising anti-inflammatory activity have therapeutic potential against inflammatory disorders.

## 1. Introduction

Inflammation is the body’s defense process in response to infection, disease, and injury [1]. During inflammation, reactive oxygen species (ROS) could be generated as a result of the host’s inflammatory response to pathogens [2]. The inflammatory process is associated with the release of pro-inflammatory cytokines [2]. Furthermore, several serious infections, such as the infection caused by SARS-CoV-2, are usually associated with elevated levels of several cytokines, including interleukins IL6, IL8 and TNFα [3], resulting in a potential cytokine storm associated with the disease’s severity [4,5]. The inflammatory response is clinically manifested by severe acute respiratory distress syndrome and disseminated intravascular coagulation [5]. Various naturally occurring plant-derived bioactive molecules have been well described as anti-oxidative, anti-inflammatory, and immune-modulatory agents, and could likely be used as potential therapies for the coronavirus disease of 2019 “COVID-19” [6,7]. Natural remedies have been used to treat inflammation in coronavirus disease and they have significantly reduced the length of hospital stay, the corticosteroid-mediated side effects in patients, and the progression of the severe disease [8,9].

*Datura*, a genus of flowering medicinal herbs from the Solanaceae family, has shown several medicinal properties [10]. *Datura stramonium* “*D. stramonium*”, widely distributed in the world, is native to deserts of the North American Southwest, Central and South America, Europe, Africa, and Asia [11]. Various indigenous communities have extensively used *D. stramonium* for medicinal purposes for centuries. In traditional medicine, various parts of the plant are used to treat a range of ailments, including respiratory disorders, gastrointestinal problems, skin diseases, and as an antalgic agent. *Datura stramonium* has been extensively studied for its potential medicinal uses. Several researches have shown that the plant contains compounds with anti-inflammatory, analgesic, and antispasmodic properties [12]. It is also known for its larvicidal effects against red flour beetles (*Tribolium castaneum*) and its mosquito-repellent activities [10]. These multiple biological activities are related to a broad range of secondary metabolites. In fact, it has been shown that *D. stramonium* contains steroids, phenolic compounds, flavonoids, alkaloids, tannins, carbohydrates, and proteins [10,12].

Withanolides are a group of structurally diverse C28 steroidal lactones with an ergostane-based skeleton. They are highly oxygenated natural products and the oxidation of different skeleton sites is responsible for the structure variation of withanolides [13]. They are present in several genera of Solanaceae such as *Acnistus*, *Datura*, *Dunalia*, *Jaborosa*, *Physalis,* and *Withania* [14]. In recent years, withanolides were devoted substantial consideration due to their versatile biological properties such as their antitumor [15], anti-inflammatory [16], immunosuppressive [17], and chemoprevention [18] properties.

*Datura stamonium* is largely known as a medicinal plant and as a source of anti-inflammatory agents [19]. However, little is known regarding the chemical composition of anti-inflammatory compounds and their inhibitory effect on pro-inflammatory cytokines.

The present study tackled this knowledge gap by assessing the antioxidant, antimicrobial, and anti-inflammatory properties of *D. stramonium* flower extract. Ultimately, the final goal was to identify *D. stramonium* anti-inflammatory compounds isolated from the flower extract and to report their molecular docking simulation with pro-inflammatory cytokines. *D. stramonium* flower extract and identified withanolide compounds could be considered anti-inflammatory candidates to alleviate inflammatory disorders associated with bacterial infections.

## 2. Results and Discussion

### 2.1. Extraction Yields and Quantification of Total Phenolic, Flavonoid, and Tannin Contents

The extraction yields obtained by the methanolic extraction of different aerial parts of *D. stramonium* (flower, seeds, and leaves) showed that the leaf extract represented the highest yield (12.87 ± 0.38%) followed by seed and flower extracts with extraction yields of 11.82 ± 0.22% and 10.52 ± 0.53%, respectively. The total phenol content of the different *D. stramonium* aerial part extracts is shown in Table 1. The seed extract showed the highest polyphenol content (219.65 ± 10.31 mg GAE/g DW, *p* < 0.05) followed by the flower extract (150.43 ± 3.08 mg GAE/g DW) (Table 1). However, the leaf extract showed the lowest amount of polyphenols with only 133.05 ± 1.96 mg GAE/g DW. Flavonoid and flavonol contents in *D. stramonium* aerial parts ranged from 65.53 to 84.79 mg CE/g DW and from 51.31 to 76.21 mg RE/g DW, respectively. The seed extract contained the highest amount of flavonoids with 84.79 ± 3.62 mg CE/g DW (*p* < 0.05). However, the leaf and flower extracts exhibited a reduced flavonoid content of 79.20 ± 0.39 and 65.53 ± 2.22 mg CE/g DW, respectively. The leaf extract exhibited the highest amount of flavonol (76.21 ± 0.3 mg RE/g DW) (*p* < 0.05), whereas the flower extract showed the lowest amount of 51.31 ± 0.68 mg RE/g DW. Condensed tannin was also recorded, and it was observed that the seed extract presented the highest content of tannins with 91.12 ± 2.32 mg CE/g DW. The flower extract, in turn, showed the lowest amount with only 16.75 ± 1.16 mg CE/g DW (*p* < 0.05). It was previously reported that the total phenolic and flavonoid contents are dependent on the *Datura* species and *Datura* organs [20,21]. In fact, *Datura innoxia* leaf extract contained the highest phenolic content compared to seed extract [22]. However, *Datura metel* seed extract exhibited a higher quantity of total phenolic content compared with *Datura metel* leaf extract.

### 2.2. Antioxidant Properties of Crude Extracts

The antioxidant assays (DPPH, ABTS and FRAP) allowed the determination of the relative antioxidant capacity of *D. stramonium* extracts. As shown in Table 2, all crude extracts of the different aerial parts of *D. stramonium* (flowers, seeds and leaves) showed potent antioxidant activity. Seed and flower extracts possessed the highest antioxidant activity compared to those of the leaf extract in the DPPH and ABTS antioxidant assays (Table 2). Seed extract showed the highest reducing power with EC_50_ 31.44 ± 1.25 µg/mL and was even comparable to that of ascorbic acid used as positive control with EC_50_ 30.75 ± 0.17 µg/mL. Leaf extract showed the lowest antioxidant activity (Table 2). Statistical analysis showed a significant difference between the antioxidant activities of the three tested extracts. The presence of a high amount of phenolic and flavonoid content agrees with the antioxidant activity shown by their extracts [20]. These compounds effectively scavenge reactive oxygen species due to the presence of phenolic hydroxyl groups in their structures. A previous work outlined the antioxidant potency of *Datura stramonium* leaf extract with an IC_50_ value of 6.7 ± 0.1 µg/mL, being more active than ascorbic acid which was used as a positive control with IC_50_ 8.9 ± 0.1 µg/mL [23]. Another study revealed that the methanolic seed extract of *D. stramonium* exhibited a low DPPH scavenging effect with an IC_50_ value of 94.87 µg/mL [24]. The differences between the antioxidant activities of *D. stramonium* extracts could be related to the differences between their phenolic compound contents. Moreover, it was previously reported that the difference in the level of antioxidant activity of *Datura innoxia* and *Datura metel* leaf and seed extracts could be associated with the level of phenolic and flavonoid contents [20,25].

### 2.3. Antimicrobial Activity

The antimicrobial activity of different aerial parts of *D. stramonium* has been assessed (Table 3). The results show that the flower extract efficiently suppressed the growth of Gram-positive bacteria, displaying the highest activity against *Enterococcus feacalis* ATCC 29212 with a minimal inhibitory concentration “MIC” value of 7.81 µg/mL. The flower extract showed moderate activity against *Staphylococcus aureus* ATCC 6538 and *Bacillus cereus* ATCC 14579 strains with a MIC value of 500 µg/mL. However, the flower extract did not exhibit antibacterial activity against Gram-negative bacteria and did not show anti-*Candida* activity against the *Candida albicans* ATCC 10231 strain. Furthermore, leaf extract exhibited moderate antibacterial activity against the Gram-positive bacterium *S. aureus*, with a MIC value of 1000 µg/mL, but was inactive against all tested Gram-negative bacteria and against *C. albicans*. However, seed extract did not show antimicrobial activity (Table 3). The antimicrobial activity of the methanolic leaf extract of *D. stramonium* was already observed against *Bacillus subtilis* and *Escherichia coli*, being more effective than that of root and stem extracts [26]. Another investigation demonstrated that the methanolic leaf extract of *D. stramonium* exhibited antibacterial activity against Gram-positive bacteria (*Staphylococcus haemolyticus*, *Staphylococcus aureus*, *Shigella dysenteriae*, and *Bacillus cereus*) and against Gram-negative bacteria, such as *Pseudomonas aeruginosa*, *Klebsiella pneumoniae*, and *Escherichia coli* at 2.5, 1.25, and 0.75 mg/mL, respectively [27]. It was suggested that the promising antibacterial activity of plant extracts against pathogenic bacterial strains may be mainly related to their chemical composition as polyphenols, flavonoids, alkaloids, saponins, glycosides, and steroids [28,29]. In fact, the steroidal, 4-[2-[[[(20*S*,22*E*)-26,27-dinorergosta-4,6,22-trien-3-yl]oxy]amino]ethynyl]-2,6-dimethylphenol isolated from the leaf ethanolic extract of *Datura metel,* has shown antibacterial activity against *Staphylococcus aureus*, *Pseudomonas aeruginosa*, *Proteus mirabis*, *Salmonella typhi*, *Bacillus subtilis* and *Klebsiella pneumonae* [30]. Bacterial infections are always associated with an inflammatory response causing tissue damage and contributing to the development of chronic diseases. The use of plant extracts with both anti-inflammatory and antibacterial properties holds potential in inhibiting bacterial growth and in reducing inflammation, and could therefore help decrease the risk of chronic diseases [31,32,33].

### 2.4. Antiviral Activity

The cytotoxicity assay of different parts of *D. stramonium* was first determined on Vero cells and CC_50_ values of 240.54 ± 0.52 µg/mL, 225.27 ± 0.38 µg/mL and 81.35 ± 0.72 µg/mL were recorded for flower, leaf and seed extracts, respectively. Subsequently, the antiviral activity of *D. stramonium* extracts against Herpes simplex virus type 2 (HSV-2) and coxsackie virus type B3 (CVB-3) was assessed at concentrations CC_50_/10 corresponding to 24 µg/mL, 22.5 µg/mL and 8 µg/mL, respectively, as previously recommended [34]. At these concentrations, no inhibitory effect against Herpes simplex virus type 2 (HSV-2) and coxsackie virus type B3 (CVB-3) was found for the tested extracts compared to the virus control. Few studies highlighted the antiviral potential of *Datura*. Previous reports showed the antiviral activity of *Datura* fruit and seed extracts against rabies virus with low IC_50_ values of 2.5 mg/mL and 1.25 mg/mL, respectively, and with a low selectivity index (CC_50_/IC_50_) ranging from two to four [35]. Recently, it was observed that anisodamine, a tropane alkaloid found in plants of the Solanaceae family (among them *Datura*), suppressed SARS-CoV-2 infection in Vero E6 cells, and reduced the SARS-CoV-2 pseudovirus entry to HEK293/hACE2 cells at 100 mg/L [36]. However, no antiviral activity against HSV-2 and CVB-3 viruses has been reported for *Datura*.

### 2.5. Nitric Oxide Inhibitory Activity and Cytotoxicity Effect

The inhibitory effect of *D. stramonium* extracts on pro-inflammatory mediator production was determined by measuring the level of nitric oxide “NO” in Raw246.7 cells stimulated by LPS. Treatments with extracts showed a decrease in NO production in a dose-dependent manner (Figure S1A). The flower extract showed the highest inhibitory effect of NO production in macrophage cells with an IC_50_ value of 26.98 ± 0.42 µg/mL. However, seed and leaf extracts were less active and moderately inhibited NO production with IC_50_ values of 96.04 ± 7.32 and 101.12 ± 6.59 µg/mL, respectively (Table 4). The cell viability analysis of Macrophage RAW 264.7 was conducted by exposure to two-fold dilutions of *D. stramonium* extracts ranging from 31.25 to 250 µg/mL (Figure S1B). Results showed that the viability of the cells decreased in a dose-dependent manner. In fact, the flower and leaf extracts showed a low cytotoxic effect at concentrations from 31.25 to 125 µg/mL with cell viability ranging from 76.33 ± 0.14% to 57.29 ± 0.52% and from 68.16 ± 0.29% to 55.04 ± 0.14%, respectively. However, seed extract was more cytotoxic with cell viability from 55.49 ± 0.29% to 39.44 ± 0.36% at concentrations from 31.25 to 125 µg/mL. At the higher concentration, corresponding to 250 µg/mL, all *D. stramonium* extracts were cytotoxic and cell viability was about 18.84 ± 0.29%, 25.33 ± 0.25%, and 12.09 ± 0.43% for flower, leaf, and seed extracts, respectively. The 50% cytotoxic concentration (CC_50_) was also determined (Table 4). Seed extract exhibited the highest cytotoxicity effect on macrophage cells with a CC_50_ value of 59.71 ± 0.44 µg/mL. Flower and leaf extracts were less cytotoxic with CC_50_ values of 142.54 ± 4.15 µg/mL and 149.05 ± 0.43 µg/mL, respectively. It was previously noticed that the seed extract of *D. stramonium* is the most toxic extract compared to other parts of the plant due to the high amount of alkaloid content [37]. To determine whether *D. stramonium* extracts showed a NO inhibitory effect with low toxicity on macrophage cells, the selectivity index (SI) was recorded (Table 4). Results indicated that the flower extract had the highest SI of 5.28, indicating that the flower extract showed a NO inhibitory effect without affecting the viability of macrophage cells. However, leaf and seed extracts showed an SI of 1.47 and 0.62, respectively, indicating that both extracts showed a NO inhibitory effect at toxic concentrations. Previous studies have shown that *D. stramonium* ethyl acetate leaf extract exhibited a NO scavenging effect with an IC_50_ value of 7.625 ± 0.551 µg/mL [38]. The toxicity effect of the extract has also been assessed, showing no significant cytotoxicity against isolated normal human lymphocytes and against macrophage isolated from rat peritoneum, even at the highest concentration (20 µg/mL) [38]. It was also noticed that *D. stramonium* leaf and seed extracts exhibited potent anti-inflammatory activity as they inhibit platelet aggregation and it was suggested that the plant could be a source of new anti-inflammatory agents [39].

### 2.6. Heatmap Analysis

**Figure 1:**
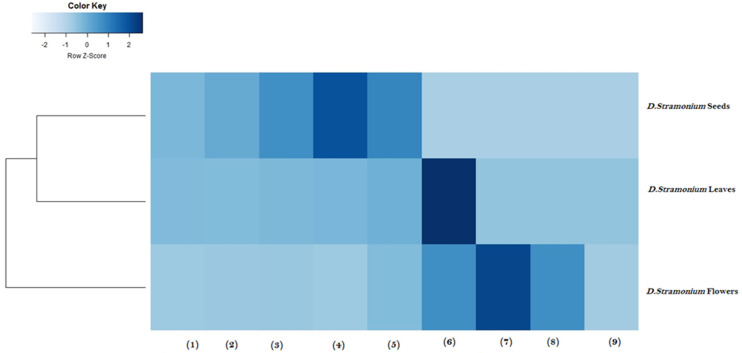
Heatmap analysis of *D. stramonium* flower, leaf and seed extracts. The clustering of samples is presented to select the best extract with large biological activities and with low cytotoxicity effect. (1) DPPH (IC_50_), (2) FRAP (EC_50_), (3) ABTS (IC_50_), (4) anti-inflammatory activity (IC_50_), (5) cytotoxicity effect (LC_50_), (6) MIC against *S. aureus* ATCC 6538, (7) MIC against MRSA, (8) MIC against *Bacillus cereus* ATCC 14579, and (9) MIC against *Enterococcus feacalis* ATCC 29212.

Heatmap analysis was performed to cluster aerial parts of *D. stramonium* extracts according to their biological activities and their cytotoxic effects (Figure 1). Clustering analysis was carried out using Euclidean distance to identify clusters with similar values and with a similar color. A low similarity was observed between *D. stramonium* flower extract and *D. stramonium* leaf and seed extracts. Hence, *D. stramonium* flower extract with potent antimicrobial, antioxidant, and anti-inflammatory activities and with less cytotoxic effects against macrophage RAW 264.7 cells was clustered in Cluster I. However, leaf and seed extracts of *D. stramonium* were grouped in cluster II with lower antimicrobial and anti-inflammatory activities and greater cytotoxic effect. Taken together, heatmap analysis demonstrated that *D. stramonium* flower extract is the most potent extract with potential biological activities and with a low cytotoxic effect. Heatmap cluster analysis is a popular strategy to cluster medicinal plants based on their biological activities [40]. It is an efficient tool to facilitate the comparison between plant extracts [41]. Benabderrahim and collaborators [22] have used heatmap clustering of plant extracts based on phenol compound composition and antioxidant activity to identify two main clusters.

### 2.7. Purification and Characterization of Anti-Inflammatory Agents

**Figure 2:**
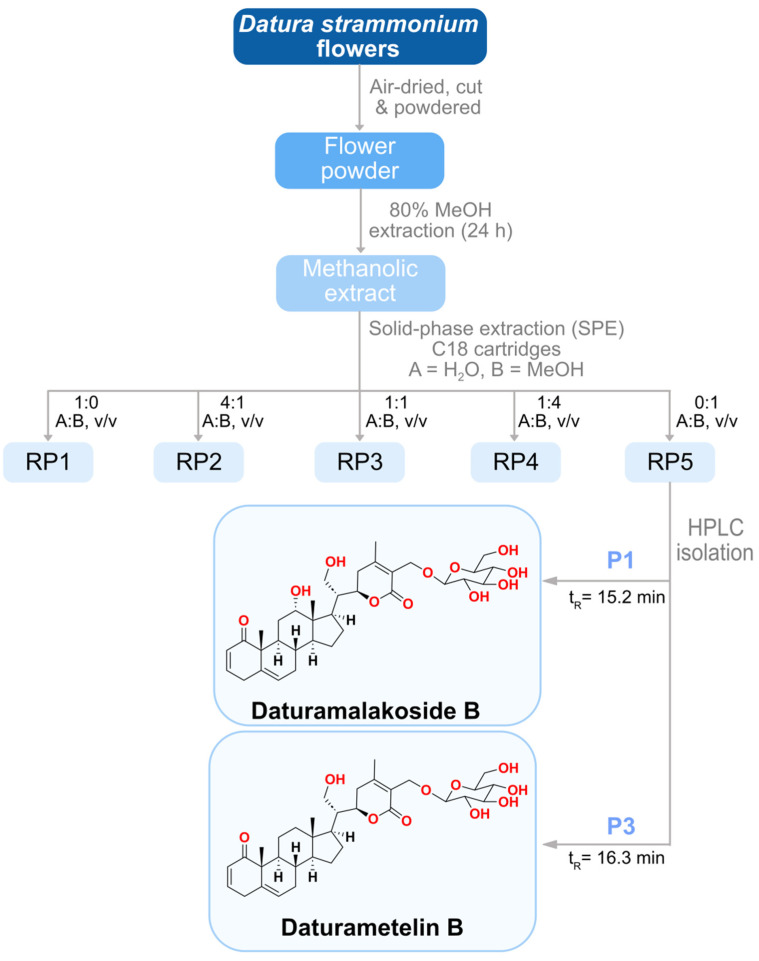
Schematic representation of the extraction and the isolation process of 12α-hydroxydaturametelin B (daturamalakoside B) and daturametelin B from *D*. *stramonium* flowers.

**Figure 3:**
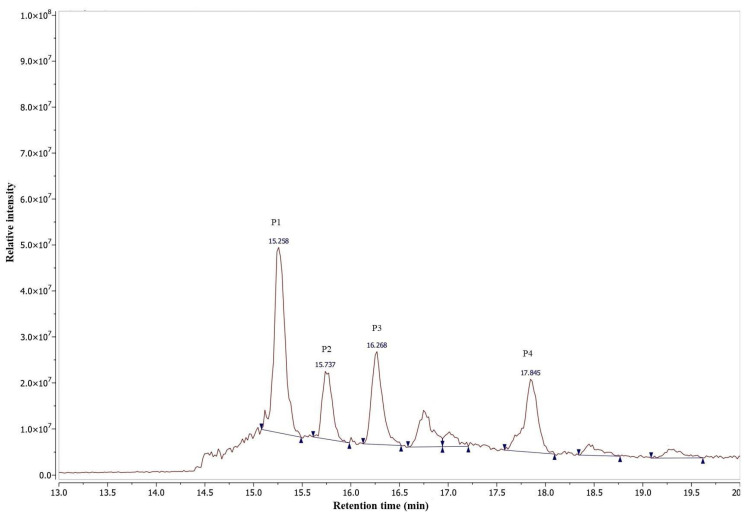
RP-HPLC chromatogram of RP5 fraction of *D*. *stramonium* flower extract. P1, P2, P3, and P4 subfractions with anti-inflammatory activity.

**Figure 4:**
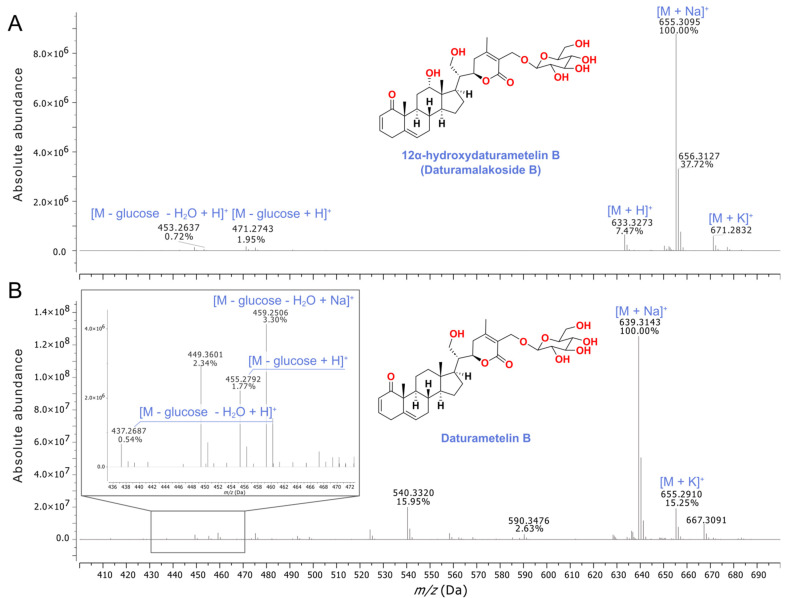
(+)–HR-ESI mass spectra of the compounds isolated from P1 (**A**) and P3 (**B**) subfractions assigned to 12α-hydroxydaturametelin B (daturamalakoside B) and daturametelin B, respectively.

*D. stramonium* flower extract with potent anti-inflammatory activity and a low cytotoxic effect was further fractionated using a reverse phase solid phase extraction (RP-SPE) to isolate the active molecules. Five fractions in total were collected as follows: 100% H_2_O (RP1, 5.6 g), 25% MeOH (RP2, 1.3 g), 50% MeOH (RP3, 1.4 g), 75% MeOH (RP4, 0.5 g) and 100% MeOH (RP5, 0.5 g) (Figure 2). RP5 fraction, eluted with 100% MeOH and collected from the SPE of the flower extract using a Sep-Pak C18 cartridge, showed an anti-inflammatory effect with an IC_50_ value 25.05 ± 0.32 µg/mL, comparable to that observed for the flower crude extract (Table 4). The cytotoxic effect was also determined for RP5 with a CC_50_ value of 125.58 ± 0.60 µg/mL and with a selectivity index of 5.01. RP5 fraction was further purified by RP-HPLC and four sub-fractions were collected: P1 (5.3 mg), P2 (2.3 mg), P3 (2.7 mg), and P4 (2.0 mg) eluted at retention times 15.258, 15.737, 16.268 and 17.854 min, respectively (Figure 3). The evaluation of the anti-inflammatory activity of subfractions P1–P4 showed that all of them were active. Subfractions P1, P2, and P3 exhibited potent anti-inflammatory activity with IC_50_ values of 6.77 ± 0.03, 7.16 ± 0.2, and 7.55 ± 0.09 µg/mL, respectively, while subfraction P4 with an IC_50_ value of 14.48 ± 0.13 µg/mL exhibited the lowest anti-inflammatory activity. The evaluation of their cytotoxic effect was also performed. Subfractions P1 and P3 showed a selectivity index of 5.74 and 7.37, respectively, and they were less cytotoxic than P2 and P4, with a selectivity index of 4.16 and 3.37, respectively (Table 4). For this reason, we focused our attention on subfractions P1 and P3. They were analyzed by HR-ESI-MS in a positive ion mode (Figure 4). Mass spectrum (MS) analysis of subfraction P1 showed a [M + Na]^+^ ion at *m*/*z* 655 with major fragments at *m*/*z* 471 [M − glucose + H]^+^ and 453 [M − glucose − H_2_O + H]^+^ (Figure 4A). Similarly, MS analysis of subfraction P3 displayed [M + Na]^+^ ion at *m*/*z* 639 (Figure 4B) with a similar fragmentation partner observed for P1.

### 2.8. Identification and Structure Elucidation of the Anti-Inflammatory Agents

The molecular formula of the compound isolated from subfraction P1 was determined as C_34_H_48_O_11_ by HR-ESI-MS with [M + Na]^+^ ion at *m*/*z* 655.3095 (calculated for C_34_H_48_O_11_Na^+^, *m*/*z* 655.3088, Δ 1 ppm). Major fragments, [M−glucose + H]^+^ at *m*/*z* 471.2743 (calculated for C_28_H_39_O_6_^+^, *m*/*z* 471.2741, Δ 0.4 ppm) and [M−glucose−H_2_O + H]^+^ at 453.2637 (calculated for C_28_H_37_O_5_^+^, *m/z* 453.2635, Δ 0.4 ppm), were detected in its MS (Figure 4A). The ^1^H and ^13^C-NMR spectral data in CD_3_OD (Figures S2–S8) of this compound (Table S1) matched those reported for (20*R*,22*R*,24*R*)-12α,21,27-trihydroxy-1-oxowitha-2,5,24-trienolide-27-*O*-ß-d-glucopyranoside, also known as 12α-hydroxydaturametelin B (daturamalakoside B). On the other hand, a molecular formula of C_34_H_48_O_10_ was assigned to the compound isolated from subfraction P3 with [M + Na]^+^ ion at *m*/*z* 639.3143 (calculated for C_34_H_48_O_10_Na^+^, *m*/*z* 639.3140, Δ 0.5 ppm). Major fragments, [M – glucose − H_2_O + Na]^+^ at *m*/*z* 459.2506 (calculated for C_28_H_36_NaO_4_^+^, *m*/*z* 459.2506, Δ 0 ppm), [M − glucose + H]^+^ at *m*/*z* 455.2792 (calculated for C_28_H_39_O_5_^+^, 455.2792, Δ 0 ppm), and [M – glucose − H_2_O + H]^+^ at 437.2687 (calculated for C_28_H_37_O_4_^+^, 437.2686, Δ 0.2 ppm), were also observed in its HR-ESI-MS (Figure 4B). The ^1^H and ^13^C-NMR spectral data in CD_3_OD (Figures S9–S14) of this compound (Table S1) matched those reported for daturametelin B. The identified compounds daturametelin B and 12α-hydroxydaturametelin B/daturamalakoside B were previously isolated from Tunisian *Datura metel* L. leaves [42] and *Datura inoxia* Mill. leaves [43]. To our knowledge, this is the first report outlying the isolation of daturametelin B and 12α-hydroxydaturametelin B (daturamalakoside B) from *D. stramonium* flower extract as anti-inflammatory agents. Withanolides are a group of naturally occurring steroidal compounds with an ergostane skeleton [16,44] produced by a variety of Solanaceae plants and they often exhibit anti-inflammatory properties [45]. For example, nine new withanolides, named daturafolisides A–I, along with six known compounds (22*R*)-27-hydroxy-7α-methoxy-1-oxowitha-3,5,24-trienolide-27-*O*-β-D-glucopyranoside, daturataturin A, daturametelin J, daturataurin B, baimantuoluoside B, and 12-deoxywithastramonolide, were isolated from the leaves of *Datura metel* L. [14]. These compounds have also shown different anti-inflammatory potentials using LPS-stimulated Raw 264.7 murine macrophages depending on the nature and the structure of the active compound. On the other hand, a previous study reported the isolation of three lactones (daturalactone, 12-deoxywithastramonolide, and daturilin) from *D. stramonium* leaves, showing that they significantly lowered the levels of NO and pro-inflammatory cytokines, indicating their anti-inflammatory activity [19]. Furthermore, steroids and withanolides isolated from *D. metel* roots were reported to possess anti-inflammatory activity and could inhibit NO production with IC_50_ values < 100 µM where compounds showed different degrees of NO inhibition according to their compositions [46]. No significant cytotoxicity effect was detected when cells were treated with steroid and withanolide compounds at concentrations up to 100 μM. Moreover, withanolides isolated from the ethanolic extract of *D. metel* leaves inhibited NO production by LPS-stimulated RAW 264 cells with IC_50_ values of 13.74 and 1392 µM [47], respectively. Other compounds isolated from *D. metel* leaf extract, i.e. daturafolisides A and B, baimantuoluoside B and 12-deoxywithastramonolide, exhibited significant inhibition of nitrite production with IC_50_ values of 20.9, 17.7, 17.8, and 18.4 μM, respectively. However, daturafolisides C, D, and F and daturataurin B presented moderate inhibitory activities with IC_50_ values of 59.0, 52.8, 71.2, and 53.1 μM, respectively [14].

### 2.9. Molecular Docking

**Figure 5:**
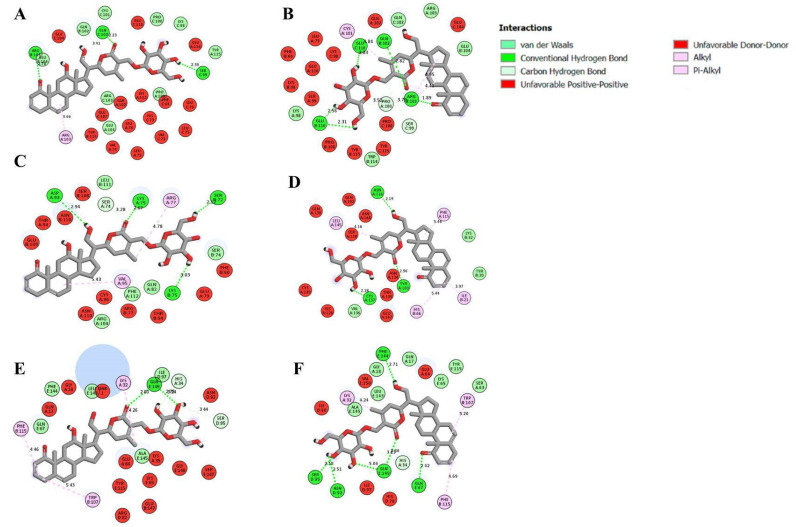
Docked poses of TNF-α with (**A**) 12α-hydroxydaturametelin B (daturamalakoside B), (**B**) daturametelin B, TNFR1 with (**C**) 12α-hydroxydaturametelin B (daturamalakoside B), (**D**) daturametelin B and TNF-α-TNFR1complex with (**E**) 12α-hydroxydaturametelin B (daturamalakoside B), (**F**) daturametelin B. The structural graphics were generated using Discovery Studio 2021.

The isolated anti-inflammatory molecules 12α-hydroxydaturametelin B and daturametelin B were tested by molecular docking analysis with pro-inflammatory cytokines interleukin-6 “IL-6”, tumor necrosis factor-α “TNF-α”, their receptors (TNFR1 and IL-6R) and both TNFα-TNFR1 and IL-6-IL-6R complexes which are essential mediators in the inflammation process. Molecular docking was investigated in order to block these pro-inflammatory cytokines. This process allows studying the eventual interaction of the identified active molecules 12α-hydroxydaturametelin B (daturamalakoside B) and daturametelin B with the active sites of TNFα, TNFR1, and the TNFα-TNFR1 complex. The interaction with the active sites of IL-6, IL-6R, and the IL-6-IL-6R complex was also investigated. Table S2 gives the details of the ligands selected for molecular docking and Figure S15 shows the molecular docking interaction of target proteins with ligands. Table 5 and Table 6 show molecular docking data represented in terms of binding energy (ΔG) in kcal/mol for the studied molecules 12α-hydroxydaturametelin B (daturamalakoside B) and daturametelin B and selected receptors involved in inflammatory activity. The results show that 12α-hydroxydaturametelin B (daturamalakoside B) and daturametelin B are binding to all selected anti-inflammatory proteins to some extent. Daturametelin B has an efficient binding energy with TNFα of −11.40 kcal/mol. This interaction requires five conventional hydrogen bonds, three alkyl bonds, two carbon–hydrogen bonds and five Van der Waal interactions (Figure 5). 12α-hydroxydaturametelin B (daturamalakoside B) showed less binding energy with TNFα active site amino acids at a −10.70 kcal/mol binding energy. This interaction involved three conventional hydrogen bonds, one alkyl bond, one carbon–hydrogen bond and nine Van der Waals interactions. A previous study outlined the interaction of saponin molecules with TNF-α and less binding energy was recorded in the range of −7.1 to −9.1 kcal/mol [48].

12α-hydroxydaturametelin B (daturamalakoside B) interacts with active site amino acids of TNFR1 with −10.30 kcal/mol binding energy and these interactions have involved four conventional hydrogen bonds, two alkyl bonds, one carbon–hydrogen bond and five Van der Waals interactions (Figure 5). Daturametelin B binds to TNFR1 outside the catalytic site. In the case of TNFα-TNFR1 complex interaction, 12α-hydroxydaturametelin B (daturamalakoside B) binds with a −9.70 kcal/mol binding energy. This interaction involved one conventional hydrogen bond, one alkyl bond, two Pi-alkyl bonds, two carbon–hydrogen bonds and five Van der Waals interactions. Similarly, the binding energy was recorded for the TNFα-TNFR1 complex–daturametelin B interaction (−9.60 kcal/mol). This interaction involved six conventional hydrogen bonds, two alkyl bonds, one Pi-alkyl, one carbon–hydrogen bond and seven Van der Waals interactions (Figure 5). Less binding energy was recorded for saponin molecules with TNF-α in the range of −7.1 to −9.1 kcal/mol [48]. Previous studies have reported the interaction of small molecules with TNF-α or TNFR1, where they inhibited TNF-α and TNFR1 binding and regulated related signal pathways [1,49,50,51]. Hence, the identification of TNF-α, TNFR1 and TNFα-TNFR1 complex inhibitors could be an alternative strategy to treat inflammation diseases [52].

**Figure 6:**
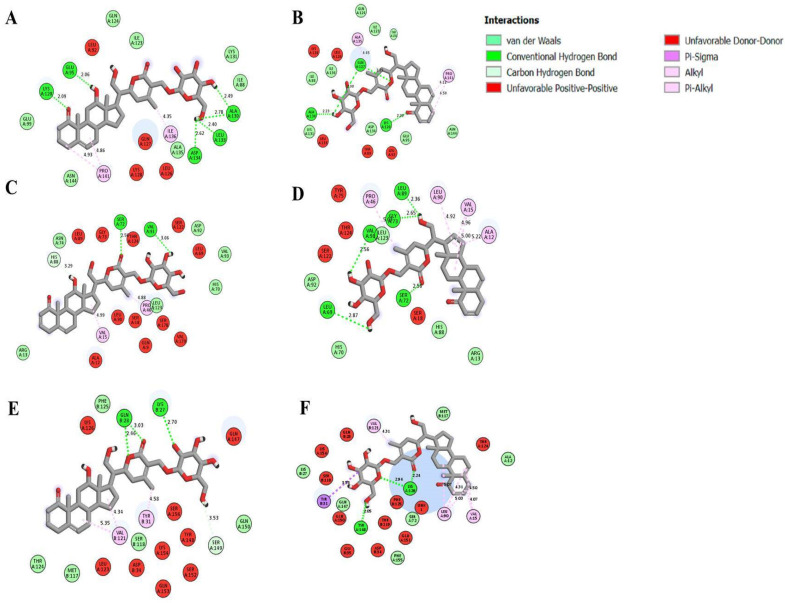
Docked poses of IL-6 with (**A**) 12α-hydroxydaturametelin B (daturamalakoside B), (**B**) daturametelin B, IL-6R with (**C**) 12α-hydroxydaturametelin B (daturamalakoside B), (**D**) daturametelin B and IL-6-IL-6R complex with (**E**) 12α-hydroxydaturametelin B (daturamalakoside B), (**F**) daturametelin B. The structural graphics were generated using Discovery Studio 2021.

The active compounds 12α-hydroxydaturametelin B (daturamalakoside B) and daturametelin B were also docked into the active site of IL-6, IL-6R, and the IL-6-IL-6R complex (Table 6). 12α-hydroxydaturametelin B (daturamalakoside B) interacts with the active site of IL-6 with a −7.40 kcal/mol binding energy. This interaction involved two conventional hydrogen bonds, one alkyl bond and two Van der Waals interactions (Figure 6). Daturametelin B also interacted with the active site of IL-6 with a −7.10 kcal/mol binding energy and this interaction involved one conventional hydrogen bond, one alkyl bond and two Van der Waals interactions (Figure 6). Furthermore, 12α-hydroxydaturametelin B (daturamalakoside B) demonstrated a strong binding energy of −9.20 kcal/mol with the IL-6R active site. This interaction involved two conventional hydrogen bonds, one alkyl bond, and one Van der Waals interaction (Figure 6). Daturametelin B interacted with IL-6R with a binding energy of −9.30 kcal/mol in the active site. This interaction required three conventional hydrogen bonds, two alkyl bonds and two Van der Waals interactions (Figure 6). The interaction with IL-6-IL-6R complex was also conducted. 12α-hydroxydaturametelin B (daturamalakoside B) interacted with the complex with a −8.10 kcal/mol binding energy. This interaction involved one conventional hydrogen bond, one alkyl bond, one carbon–hydrogen bond and one Van der Waals interaction (Figure 6). A similar interaction was observed for daturametelin B and IL-6-IL-6R complex with a −8.50 kcal/mol binding energy. This interaction involved one conventional hydrogen bond, one Van der Waals interaction and one Pi-sigma bond in the active site (Figure 6). IL-6, also known as interferon β2, is a key cytokine in the regulation of inflammation and is produced in response to infections [53]. It is also involved in the pathogenesis of rheumatoid arthritis [54]. It was previously shown that quercetin, catechin, and gallic acid from *Satureja nepeta* extract exhibited a strong affinity with IL-6R with binding energy values of −7.1, −6.1, and −5.3 kcal/mol, respectively. It was suggested that the interaction with the active site of IL-6R could be a suitable mechanism for the inhibition of IL-6 by *S. nepeta* polyphenols [53]. It was recently noticed that genistein showed an excellent binding energy (−10.59 kcal/mol) with IL-6/IL-6Rα of the STAT3 pathway and could therefore inhibit the IL-6/STAT3 pathway by blocking the interaction of IL-6 with IL-6R [55].

## 3. Material and Methods

### 3.1. Plant Material

The plant material of *Datura stramonium* (leaves, seeds, and flowers) was collected in April during the flowering stage from Turki, Grombalia, the North East region of Tunisia. The zone is located between 36°34′27.7″ N of latitude and 10°31′00.2″ E of longitude. Identification of the plant was carried out by Pr. A. Smaoui (Biotechnology Center of Borj Cedria) and a voucher specimen (Dst-BC 91-200) was deposited in the herbarium of the Biotechnology Center of Borj Cedria, Tunisia.

### 3.2. Preparation of Crude Extracts

*D. stramonium* leaves, flowers, and seeds were separated, air-dried, cut into small pieces, and powdered. The plant material (10 g) was extracted with 100 mL methanol (80%) for about 24 h at room temperature. Extracts were filtered and concentrated to dryness under reduced pressure using a rotary evaporator (Buchi, R200, Flawil, Switzerland). The resulting mixtures were suspended in deionized water and were stored at −20 °C for further investigation.

### 3.3. Determination of Total Phenolic Contents

Total phenolic content was determined using a modified Folin-Ciocalteu colorimetric method [56]. Briefly, samples were added to 500 µL of distilled water and 125 µL of Folin-Ciocalteu reagent (FCR) and then allowed to stand for 6 min in the dark at room temperature. A total of 1250 µL of a 7% sodium carbonate (NaCO_3_) aqueous solution was then added. Samples were incubated for 90 min at room temperature and the absorbance was measured at 760 nm using a UV-visible spectrophotometer (Synergy HT, BioTek, Bad Friedrichshall, Germany). The total phenolic content was expressed in milligrams of gallic acid equivalent per gram of dry extract (mg GAE/g DW) using the gallic acid calibration curve. All the samples were prepared in triplicate.

### 3.4. Determination of Total Flavonoid Content

Total flavonoid content was determined using the aluminum chloride colorimetric method as previously described [56]. Briefly, sample extracts were mixed with 5% NaNO_2_ and 10% AlCl_3_ solutions and incubated for 5 min at room temperature. A solution of NaOH 1M was then added. The mixture was further incubated for 15 min and the absorbance was measured at 510 nm using a UV-visible spectrophotometer (Synergy HT, BioTek). Catechin was used as a standard and results were expressed in milligrams of catechin equivalent per gram of dry extract (mg CE/g DW). All samples were analyzed in triplicate.

### 3.5. Determination of Condensed Tannin Content

Condensed tannins were determined by the vanillin method according to the method of Julkunen-Titto [57]. Samples were added to the 4% vanillin/methanol solution. Then, a volume of 750 μL of concentrated hydrochloric acid (HCl) was added. The mixture was allowed to stand for 20 min and the absorbance was measured at 500 nm using a UV-visible spectrophotometer against methanol as a blank. Catechin was used as a standard. Samples were analyzed in triplicate and condensed tannin was expressed in milligrams of catechin equivalent per gram of dry extract (mg CE/g DW).

### 3.6. Determination of Antioxidant Activity

#### 3.6.1. 2,2-Diphenyl-1-picrylhydrazyl (DPPH) Radical Scavenging Assay

The DPPH radical scavenging potential of extracts is based on the reduction of the purple-colored DPPH into pale yellow hydrazine according to the method described [58]. Tested extracts were added to 500 μL of 0.2 mM DPPH–methanol solution. After incubation in the dark for 30 min at room temperature, the absorbance was determined at 517 nm. The percentage inhibition of free radical DPPH was calculated according to the following formula:

DPPH inhibition (%) = [(A_blank_ − A_sample_)/A_blank_] ×100

where A_blank_ is the absorbance of the control and A_sample_ is the absorbance of the plant extract. Butylated hydroxytoluene (BHT) was used as a positive control. The concentration that caused 50% inhibition of DPPH (IC_50_) was also calculated.

#### 3.6.2. 2.2′-Azino-bis-3-ethylbenzthiazoline-6-sulphonic Acid (ABTS) Radical Scavenging Assay

The antioxidant activity of the tested samples was also evaluated against the free radical ABTS^·+^ according to Khan et al. (2012) [59]. The ABTS solution at a 7 mM concentration was oxidized by the addition of a 2.45 mM potassium persulfate solution. The solution was kept overnight in the dark to yield ABTS radical cations. Then, 1.5 mL of the solution was mixed with 50 μL of the extract. After incubation in the dark at room temperature for 10 min, the absorbance decrease was measured at 734 nm. The percentage of inhibition and IC_50_ value were determined as previously described in the DPPH test.

#### 3.6.3. Ferric Reducing Antioxidant Power (FRAP)

The reducing power of the tested samples was measured according to the method described by El Jemli and collaborators [60]. Samples were mixed with 2500 µL of a sodium phosphate buffer (0.2 M, pH 6.6) and 2500 µL of 1% potassium ferricyanide (K_3_Fe(CN)_6_). The reaction mixture was incubated for 20 min at 50 °C in a water bath. Then, 2500 μL of trichloroacetic acid (TCA) (10%) was added to stop the reaction. After centrifugation at 3000 rpm for 10 min, 2500 μL of the supernatant was removed and was mixed with 2500 μL of distilled water and 500 μL of FeCl_3_ (0.1%). The absorbance was determined at 700 nm. A higher absorbance indicated a higher reducing power. The extract that gave 0.5 absorbance (EC_50_) was determined by plotting absorbance against extract concentration. Ascorbic acid was used as the standard.

### 3.7. Antimicrobial Activity

The antimicrobial activity of *D. stramonium* aerial parts was assessed against Gram-positive strains (*Staphylococcus aureus* ATCC 6538, methicillin-resistant *Staphylococcus aureus* strain (MRSA), *Bacillus cereus* ATCC 14579 and *Enterococcus faecalis* ATCC 29212), Gram-negative strains (*Escherichia coli* ATCC 25922, *Klebsiella pneumonia* CIP 104727, *Salmonella enteridis* DMB 560 and *Pseudomonas aeruginosa* ATCC 27853) and against the yeast strain *Candida albicans* ATCC10231. The antimicrobial activity test was performed using the broth microdilution method [61]. Serial two-fold dilutions of the crude extracts of *D. stramonium* aerial parts were performed in microplates containing the appropriate medium (LB for the antibacterial activity and sabouraud broth for the anti-yeast activity). A microbial cell suspension (10^6^ CFU/mL) was then added. After overnight incubation at 30 °C, the minimal inhibitory concentration (MIC) was determined as the lowest concentration of the extract that inhibited microorganism growth. MICs ≥ 2000 µg/mL were considered as inactive [62].

### 3.8. Antiviral Activity

The antiviral activity of *D. stramonium* aerial parts was assessed against Herpes simplex virus type 2 (HSV-2) and coxsackie virus type B3 (CVB-3) [63]. Serial two-fold dilutions of the tested samples from 7.81 µg/mL to 1000 µg/mL were initially added to the semi-confluent cancer cells (Vero cell line) in a 96-well plate. Cells without the extract were used as cell controls. After 72 h of incubation at 37 °C, the cells were trypsinized to obtain a single-cell suspension and then trypan blue solution (10%, *v*/*v*) was added. The living cells were counted and the 50% cytotoxic concentration (CC_50_) was calculated by linear regression analysis from the dose–response curve. Afterwards, samples were screened for their antiviral activity in a 96-well plate. Confluent Vero cells were infected with HSV-2 and CVB-3 with a multiplicity of infection of 0.1 and 0.001 at a concentration of CC_50_/10 and CC_50_/1000, respectively. Infected cells that were not treated with the extracts were used as virus controls. After 48 h of incubation at 37 °C, the sample concentration that inhibited virus plaque was considered as an active extract.

### 3.9. Nitrite Oxide “NO” Inhibitory Effect

Raw 264.7 cells were grown in Dulbecco’s Modified Eagle’s Medium (DMEM) and 2 × 10^6^ cells were seeded in 96-well microtiter plates and allowed to adhere overnight. Cells were then treated with different concentrations of the tested samples. After incubation at 37 °C for 1 h, LPS was added at a concentration of 1 µg/mL and the mixture was further incubated at 37 °C for 24 h in an incubator with a humidified atmosphere and 5% CO_2_. The NO level in the culture supernatant was determined using Griess reagent [64]. The absorbance was measured at 570 nm using a microplate reader (Synergy HT, BioTek). A standard solution of NaNO_2_ was used to calculate the concentration of NO. The concentration that inhibited NO production by 50% (IC_50_) was also determined. L-NAME (N-nitro-L-arginine methyl ester) was used as the positive control.

### 3.10. Cytotoxicity Assay

The cytotoxicity effect of the samples was determined according to Essid et al., 2015 [65]. Briefly, macrophage RAW 267.4 cells were plated at 10^4^ cells/well and incubated at 37 °C with 5% CO_2_ for 24 h. Cells where then treated with different concentrations of samples and LPS for 24 h. An amount of 50 µL of 5 mg/mL of 3-(4,5-dimethylthiazol-2-yl)-2,5-diphenyltetrazolium bromide (MTT) (Sigma–Aldrich, St–Louis, MO, USA) solution was added and incubated at 37 °C for 4 h. Subsequently, 100 µL of a mixture of 50% (*v*/*v*) isopropanol and 10% (*w*/*v*) sodium dodecyl sulfate (SDS) was added to each well to dissolve the formazan crystals. After 30 min of incubation at room temperature, the absorbance was measured at 560 nm using an ELISA plate reader (Synergy HT, BioTek, Bad Friedrichshall, Germany). The lethal concentration (LC_50_) was calculated as the concentration of a given agent which is lethal to 50% of cells. The experiments were done in triplicate.

### 3.11. Extraction and Isolation of the Active Compounds

The methanolic extract of *D. stramonium* flower (10.3 g) was subjected to solid phase extraction (SPE) with a reversed-phase C18 cartridge (Thermo Scientific cartridge Hyper Sep C18, Dreieich, Germany) [66]. The elution was performed with a gradient from H_2_O to MeOH: 0%, 25%, 50%, 75%, and 100% MeOH, to afford the fractions RP1-RP5. The active fraction was separated by high-performance liquid chromatography (HPLC) (Agilent 1200 HPLC, Agilent, Frankfurt, Germany) connected to a UV-DAD detector recording at 254 nm. The analysis was carried out with an Atlantis semi-preparative C18 column (10 mm × 100 mm, 5 µm) at a flow rate of 2.5 mL/min, with a mobile phase consisting of a gradient of 0.05% formic acid in water (A) and 0.05% formic acid in acetonitrile (B). The elution gradient was performed as follows: 2 min of isocratic at 10% (B), 32 min of a gradient from 10 to 100% (B), 5 min of isocratic at 100% (B), 1 min of a gradient from 100 to 10% (B), and finally, 10 min of isocratic at 10% (B). The injected volume was 1000 µL at a concentration of 5 mg/mL.

### 3.12. Compound Characterization

The characterization of active sub-fractions was performed by high-resolution electrospray ionization mass spectrometry (HR-ESI-MS) analysis in a Qstar-QqTOF Tamdem Hybrid System with an ESI source (Applied BioSystems, Bremen, Germany). ^1^H, ^13^C, and 2D Nuclear Magnetic Resonance (NMR) spectra were recorded on a Bruker Avance 300 with a Neo Console NMR spectrometer (300 MHz for ^1^H, and 75 MHz for ^13^C) and on a Bruker Avance 500 spectrometer (500 MHz for ^1^H and 125 MHz for ^13^C). Chemical shifts (δ) are reported as parts per million (ppm) relative to CD_3_OD (δ 3.31 ppm for ^1^H NMR, δ 49.0 ppm for ^13^C NMR).

### 3.13. Liquid Chromatography–Mass Spectrometry (HPLC/MS) Analysis

UHPLC was performed on a Thermo Fisher Scientific Ultimate 3000. Separation was carried out on an Atlantis analytical column (4.6 mm × 100 mm, 5 µm) at a flow rate of 1 mL/min with a mobile phase consisting of a gradient of 0.05% formic acid in water (A) and 0.05% formic acid in acetonitrile (B). The elution gradient was performed as follows: 2 min of isocratic at 10% (B), 40 min of a gradient from 10 to 100% (B), 5 min of isocratic at 100% (B), 1 min of a gradient from 100 to 10% (B), and, finally, 9 min of isocratic at 10% (B). The injection volume was 15 µL and UV wavelength detection was set at 254 nm. MS analysis was operated on a Single Quadrupole Mass Spectrometer MSQ Thermo Fisher Scientific and the analysis was carried out in positive (ESI+) ion mode with a cone voltage of 75 V. The probe temperature was set at 575 °C, and the scan range at *m*/*z* 100–1500.

### 3.14. Molecular Docking

#### 3.14.1. Preparation of Target Proteins and Ligands

The crystal structure of the target proteins TNFα, TNFR1 receptor, TNFα–TNFR1 complex, IL-6, IL-6R and IL-6-IL-6R complex were obtained from the PDB (Protein Data Bank) (https://www.rcsb.org/, accessed on 16 December 2022): the extracellular domain of 55 kDa TNFR1 (PDB ID: 1EXT) with a resolution of 1.85 Å [67], TNFα structure (PDB ID: 1TNF) [68] with a resolution of 2.60 Å and the TNFα–TNFR1 complex protein were obtained by protein–protein docking using pyDock (JOBID: J_6374AD30186DE) with electrostatics at −18.667, desolvation at 12.230, VdW at −36.505 and relRST at 15.789. The crystal structure of the extra-cellular domains of human interleukin-6 Receptor alpha chain (IL-6R) was also measured (PDB ID: 1N26]) with a resolution of 2.40 Å [69], human interleukin-6 structure (PDB ID: 1ALU) with a resolution of 1.90 Å [70] and IL-6-IL-6R complex protein was obtained by protein–protein docking using pyDock (JOBID: J_637781CCAE854) with electrostatics at −18.241, desolvation at 4.658, VdW at 26.457 and relRST at 82.353. Active sites of the studied target proteins TNFR1, TNFα, TNFα–TNFR1 complex, IL-6R, IL-6 and the IL-6-IL-6R complex were predicted using Computed Atlas for Surface Topography of Proteins (CASTp) (http://cast.engr.uic.edu, accessed on 17 December 2022) and are presented in Figures S16 and S17. 12α-hydroxydaturametelin B (daturamalakoside B) (C_34_H_48_O_11_) and daturametelin B (C_34_H_48_O_10_) ligand structures were determined using PubChem Sketcher V2.4 and USCF Chimera 1.17 (Table S2).

#### 3.14.2. Docking Simulation

Molecular docking studies were carried out with the AutoDock 1.5.6 vina tools and all hetero atoms and crystal water molecules were removed from the target proteins with energy minimization. The docking site on the protein target was determined by establishing a grid box (Table S3). This grid must have surrounded the region of interest (active site) in the macromolecule. Docking was performed to obtain a possible conformation and orientation of the ligand at the binding site. The best conformation was chosen with the lowest binding energy. The docked structures were visualized using PyMOL version 0.99. The interaction between target proteins and ligands was also determined by Biovia Discovery Studio Visualizer v21.1.0.20298 (Biovia, D.S 2021) available online: https://discover.3ds.com/discovery-studio-visualizer-download, accessed on 13 December 2022.

### 3.15. Statistical Analysis

All experiments were carried out in triplicate and the results were expressed as mean ± standard deviation. Differences between extracts in experiments were assessed for significance using analysis of variance (ANOVA) and (one-way) Tukey test, and mean values were considered statistically significant at *p* < 0.05. The heatmap showing the antioxidant, antibacterial, anti-inflammatory and cell viability assays was constructed using RStudio Programming–Heatmap.

## 4. Conclusions

The present study demonstrated that *Datura stramonium* flower extract exhibits potent antioxidant, antibacterial, and anti-inflammatory properties. These properties could decrease the risk of chronic disease incidence. No antiviral and cytotoxicity effects were recorded. Daturametelin B and 12α-hydroxydaturametelin B (daturamalakoside B) withanolides were isolated for the first time from *D. stramonium* flower extract as the main anti-inflammatory compounds with a low cytotoxicity effect. Molecular docking simulation studies revealed that these compounds have optimal binding features with the tumor necrosis factor-α and interleukin-6 and, therefore, they have great potential to act as anti-inflammatory agents. Further studies are needed to investigate the in vivo potency of these bioactive compounds as anti-inflammatory agents and to evaluate their mechanism of action involving cyclooxygenase (COX1 and COX2) gene expression.

## Figures and Tables

**Table 1:** Total polyphenol, flavonoid, flavonols and condensed tannin contents of *D. stramonium* extracts.

Samples	Polyphenols (mg GAE/g DW)	Flavonoids (mg CE/g DW)	Flavonols (mg RE/g DW)	Condensed Tannin (mg CE/g DW)
Flower extract	150.43 ± 3.08 ^(b)^	65.53 ± 2.22 ^(b)^	51.31 ± 0.68 ^(b)^	16.75 ± 1.16 ^(c)^
Leaf extract	133.05 ± 1.96 ^(b)^	79.20 ± 0.39 ^(a)^	76.21 ± 0.30 ^(a)^	74.37 ± 0.67 ^(b)^
Seed extract	219.65 ± 10.31 ^(a)^	84.79 ± 3.62 ^(a)^	53.53 ± 1.39 ^(b)^	91.12 ± 2.32 ^(a)^

Means with different lowercase ^(a–c)^ within the same column are considered significantly different with *p* < 0.05, *n* = 3. GAE: Gallic acid equivalents, CE: cathechin equivalents, RE: rutin equivalents, DW: dry weight.

**Table 2:** Antioxidant properties of *D. stramonium* extracts.

Samples	Antioxidant Activity		
	DPPH Radical Scavenging Activity IC_50_ (µg/mL)	ABTS Assay IC_50_ (µg/mL)	Reducing Power EC_50_ (µg/mL)
Flower extract	22.82 ± 2.01 ^(b)^	52.69 ± 1.55^(b)^	41.75 ± 1.55 ^(b)^
Leaf extract	70.98 ± 0.56 ^(c)^	89.81 ± 1.31 ^(c)^	65.50 ± 3.92 ^(c)^
Seed extract	21.62 ± 1.72 ^(b)^	52.31 ± 0.69 ^(b)^	31.44 ± 1.25 ^(a)^
BHT	14.54 ± 0.30 ^(a)^	-	-
Ascorbic acid	-	28.23 ± 0.26 ^(a)^	30.75 ± 0.17 ^(a)^

Means with different lowercase ^(a–c)^ within the same column are considered significantly different with *p* < 0.05, *n* = 3.

**Table 3:** Antibacterial activity of *D. stramonium* extracts.

Microbial Strains	Antimicrobial Activity: MIC (µg/mL)		
	Leaf Extract	Flower Extract	Seed Extract
Gram-positive bacteria			
*Staphylococcus aureus* ATCC 6538	1000	500	≥2000
Methicillin Resistant *Staphylococcus aureus* (MRSA)	≥2000	1000	≥2000
*Bacillus cereus* ATCC 14579	≥2000	500	≥2000
*Enterococcus feacalis* ATCC 29212	≥2000	7.81	≥2000
Gram-negative bacteria			
*Escherichia coli* ATCC 25922	≥2000	≥2000	≥2000
*Salmonella enteritidis* DMB 560	≥2000	≥2000	≥2000
*Klebsiella pneumonia* CIP 104727	≥2000	≥2000	≥2000
*Pseudomonas aeruginosa* ATCC 27853	≥2000	≥2000	≥2000
Yeasts			
*Candida albicans* ATCC 10231	≥2000	≥2000	≥2000

**Table 4:** Nitric oxide inhibitory activity, cytotoxic effect, and selectivity index of *D. stramonium* extracts, RP5 fraction, and P1–P4 subfractions.

Samples	NO Inhibition IC_50_ (µg/mL)	Cytotoxic Effect LC_50_ (µg/mL)	Selectivity Index (SI)
Leaf extract	101.12 ± 6.59 ^(b)^	149.05 ± 0.43 ^(b)^	1.47
Seed extract	96.04 ± 7.32 ^(b)^	59.71 ± 0.44 ^(a)^	0.62
Flower extract	26.98 ± 0.42 ^(a)^	142.54 ± 4.15 ^(d)^	5.28
L-NAME	21.30 ± 0.24 ^(a)^	332.41 ± 1.7 ^(a)^	15.61
RP5 fraction	25.05 ± 0.32 ^(d)^	125.58 ± 0.60 ^(b)^	5.01
P1	6.77 ± 0.03 ^(a)^	38.9 ± 0.02 ^(e)^	5.74
P2	7.16 ± 0.02 ^(ab)^	29.83 ± 0.14 ^(f)^	4.16
P3	7.55 ± 0.09 ^(b)^	55.65 ± 0.61 ^(c)^	7.37
P4	14.48 ± 0.13 ^(c)^	48.92 ± 0.48 ^(d)^	3.37
L-NAME	21.30 ± 0.24 ^(d)^	332.41 ± 1.7 ^(a)^	15.61

Means with different lowercase ^(a–f)^ are considered significantly different with *p* < 0.05, *n* = 3. RP5: active fraction eluted with 100% MeOH from Sep-Pak C18 cartridge; P1, P2, P3 and P4: four active peaks obtained after injection of RP5 in RP-HPLC; L-NAME: N-nitro-l-arginine methyl ester used as a positive control.

**Table 5:** Molecular docking data represented in terms of binding energy in kcal/mol of target proteins with 12α-hydroxydaturametelin B (daturamalakoside B) and daturametelin B.

Receptors	Ligands	Binding Energy (kcal/mol)	Active Site Amino Acids	Interaction Type
TNF α	12α-hydroxydaturametelin B (daturamalakoside B)	−10.70	B/ARG.103, C/SER.99, C/GLN.102	Hydrogen
			A/ARG.103	Alkyl
			C/GLN.102	Carbon–Hydrogen
			A/PRO.100,A/GLU.104,A/TYR.115,B/GLN.102,B/GLU.104,C/LYS.98,C/PRO.100,C/CYS.101,C/ARG.103	Van der Waals
	Daturametelin B	−11.40	B/GLN.102,B/ARG.103, B/GLU.116, C/GLU.116	Hydrogen
			A/CYS.101,B/ARG.103	Alkyl
			A/PRO.100, C/SER.99	Carbon–Hydrogen
			A/LYS.98, A/AGR.103, B/GLU.104,B/TRP.114, C/GLN.102	Van der Waals
TNFR1	12α-hydroxydaturametelin B (daturamalakoside B)	−10.30	A/LYS.75, A/ASP.93, B/SER.72, B/LYS.75	Hydrogen
			A/ARG.77, A/VAL.95	Alkyl
			A/SER.74	Carbon–Hydrogen
			A/GLN.82,A/ARG.104, A/PHE.112, B/SER.74, B/LEU.111	Van der Waals
	Daturametelin B	−8.70		∅
TNFα-TNFR1complex	12α-hydroxydaturametelin B (daturamalakoside B)	−9.70	E/GLN.149	Hydrogen
			B/TRP.107	alkyl
			B/LYS.32, B/PHE.115	Pi-Alkyl
			A/HIS.34, D/SER.95	Carbon–Hydrogen
			D/ILE.97, E/GLN.67, E/LEU.143, E/PHE.144, E/ALA.145	Van der Waals
	Daturametelin B	−9.60	D/ASN.92, D/SER.95, E/GLN.67, E/PHE.144, E/GLN.149	Hydrogen
			A/LYS.32, B/PHE.115	Alkyl
			B/TRP.107	Pi-Alkyl
			A/HIS.34	Carbon–Hydrogen
			A/GLN.17, A/GLY.18, A/SER.63, E/LYS.65, E/TYR.115,E/LEU.143, E/ALA.145	Van der Waals

**Table 6:** Molecular docking results of 12α-hydroxydaturametelin B (daturamalakoside B) and daturametelin B with human IL-6, extra-cellular domains of human interleukin-6 Receptor alpha chain (IL-6R) and IL-6-IL-6R complex.

Receptors	Ligands	Binding Energy (kcal/mol)	Active Site Aminoacids	Interaction Type
IL-6	12α-hydroxydaturametelin B (daturamalakoside B)	−7.40	A/GLU.95, A/LYS.120	Hydrogen
			A/PRO.141	alkyl
			A/GLU.99, A/ASN.144	Van der Waals
	Daturametelin B	−7.10	A/LYS.120	Hydrogen
			A/PRO.141	Alkyl
			A/GLU.95, A/ASN.144	Van der Waals
IL-6R	12α-hydroxydaturametelin B (daturamalakoside B)	−9.20	A/SER.72, A/VAL.91	Hydrogen
			A/PRO.46	Alkyl
			A/ASP.92	Van der Waals
	Daturametelin B	−9.30	A/LEU.69, A/SER.72, A/VAL.91	Hydrogen
			A/PRO.46, A/LEU.90	Alkyl
			A/ASP.92, A/LEU.123	Van der Waals
IL-6-IL-6R	12α-hydroxydaturametelin B (daturamalakoside B)	−8.10	B/LYS.27	Hydrogen
			B/TYR.31	alkyl
			A/SER.149	Carbon–Hydrogen
			A/GLN.150	Van der Waals
	Daturametelin B	−8.50	A/TYR.148	Hydrogen
			B/TYR.31	Pi-Sigma
			B/LYS.27	Van der Waals

## Data Availability

Not applicable.

## Supplementary Materials

The following supporting information can be downloaded at: https://www.mdpi.com/article/10.3390/molecules28135195/s1, Figure S1: Effect of flower, leaf and seed extracts of *D. stramonium* on LPS induced NO production and cytotoxicity effect on macrophage RAW 264.7 cells; Figures S2–S4 and Table S1: NMR spectrum of 12α-hydroxydaturametelin B; Figures S5–S7: 1H-1H COSY spectrum, Edited-HSQC spectrum and HMBC spectrum of 12α-hydroxydaturametelin B; Figure S8: ESI (+)-HRMS spectrum of 12α-hydroxydaturametelin B; Figures S9–S11 and Table S1: NMR spectrum of daturametelin B; Figure S12 and Figure S13: Edited-HSQC spectrum and 1H-1H COSY spectrum of daturametelin B; Figure S14: ESI (+)-HRMS spectrum of daturametelin B; Figure S15: 3D illustration of TNF-α binding poses with 12α-hydroxydaturametelin B and Daturametelin B, TNFR1 with 12α-hydroxydaturametelin B and Daturametelin B, TNF-α-TNFR1complex with 12α-hydroxydaturametelin B and Daturametelin B; Figure S16: 3D illustration of IL-6 binding poses with 12α-hydroxydaturametelin B and Daturametelin B, IL-6R with 12α-hydroxydaturametelin B and Daturametelin B, IL-6–IL-6R complex with 12α-hydroxydaturametelin B and Daturametelin B; Figure S17: The active sites of TNF-α, TNFR1, TNF-α–TNFR1 complex, Human IL-6, extra-cellular domains of Human Interleukin-6 Receptor alpha chain (IL-6R) and the IL-6–IL-6R complex; Table S2: 12α-hydroxydaturametelin B and daturametelin B characteristics and structures obtained from PubChem Sketcher V2.4; Table S3: Grid box parameters selected for the target proteins.

## Sample Availability

Samples of the compounds are available from the first and corresponding authors.
