# Supplementary material for: Datura stramonium Flowers as a Potential Natural Resource of Bioactive Molecules: Identification of Anti-Inflammatory Agents and Molecular Docking Analysis
Source: Molecules. 2023 Jul 4;28(13):5195. doi: 10.3390/molecules28135195 (PMC10343631; doi:10.3390/molecules28135195)
Supplement: Supplementary file 1 [file molecules-28-05195-s001.zip › molecules-2390850-supplementary.pdf]

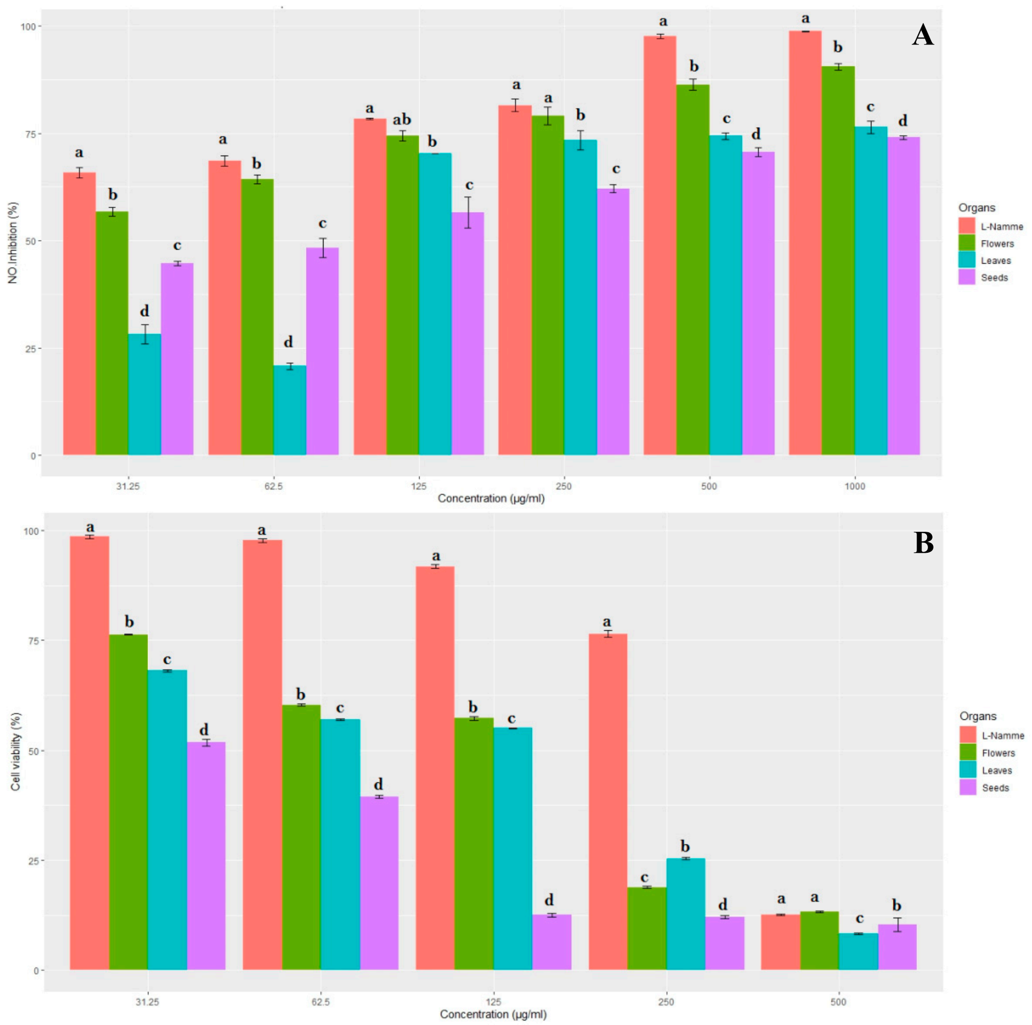

Fig. S1 Effect of flower, leaf and seed extracts of *D. stramonium* on LPS induced NO production in macrophage RAW 264.7 cells (A) and cytotoxicity effect on macrophage RAW 264.7 cells (B).Values represent the means of triplicate measurements (n = 3). Bars represent the standard deviation.

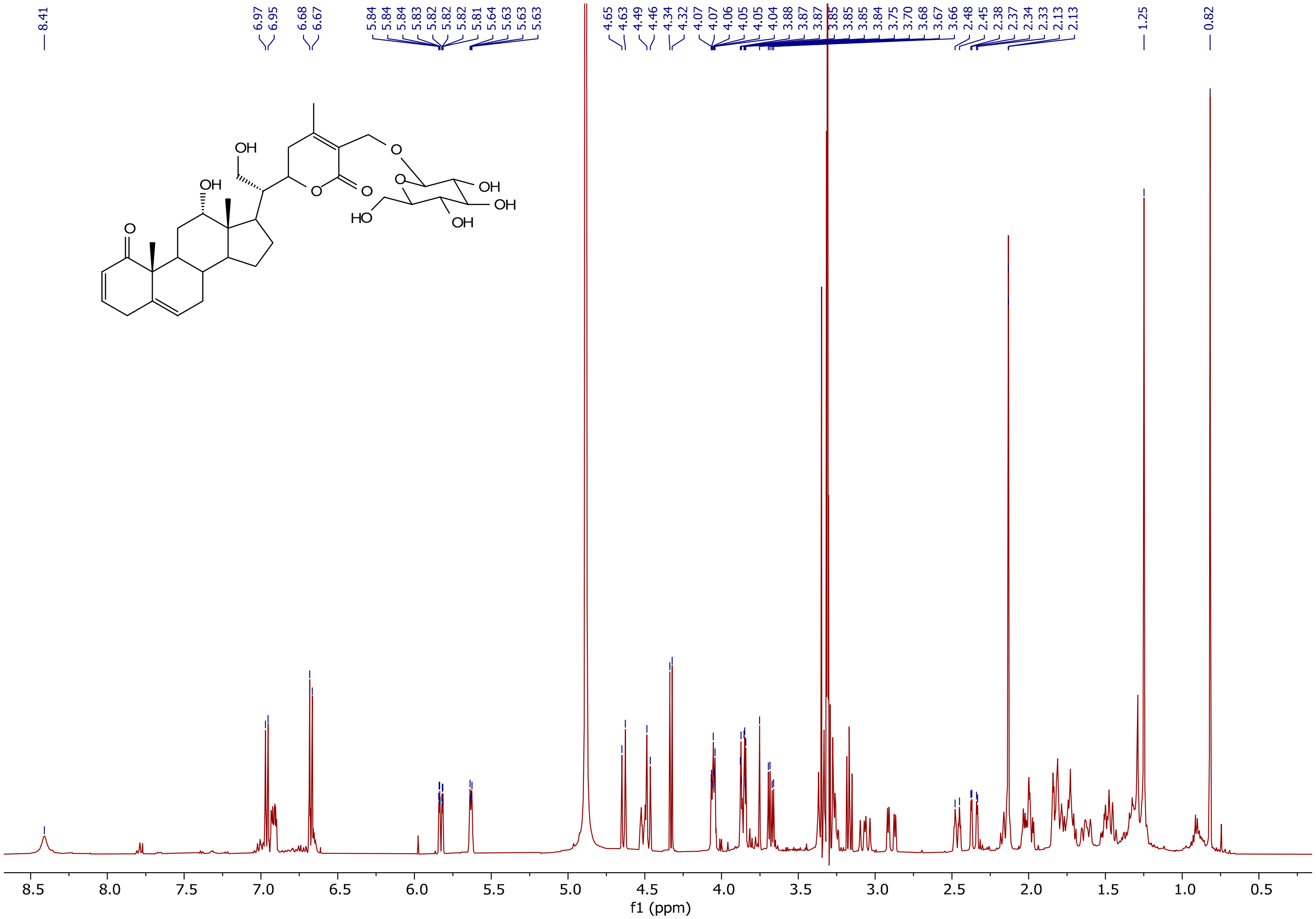

Fig. S2. ^{1}H NMR spectrum (CD_3OD, 500 MHz) of 12a-hydroxydaturametelin B (daturamalakoside B)

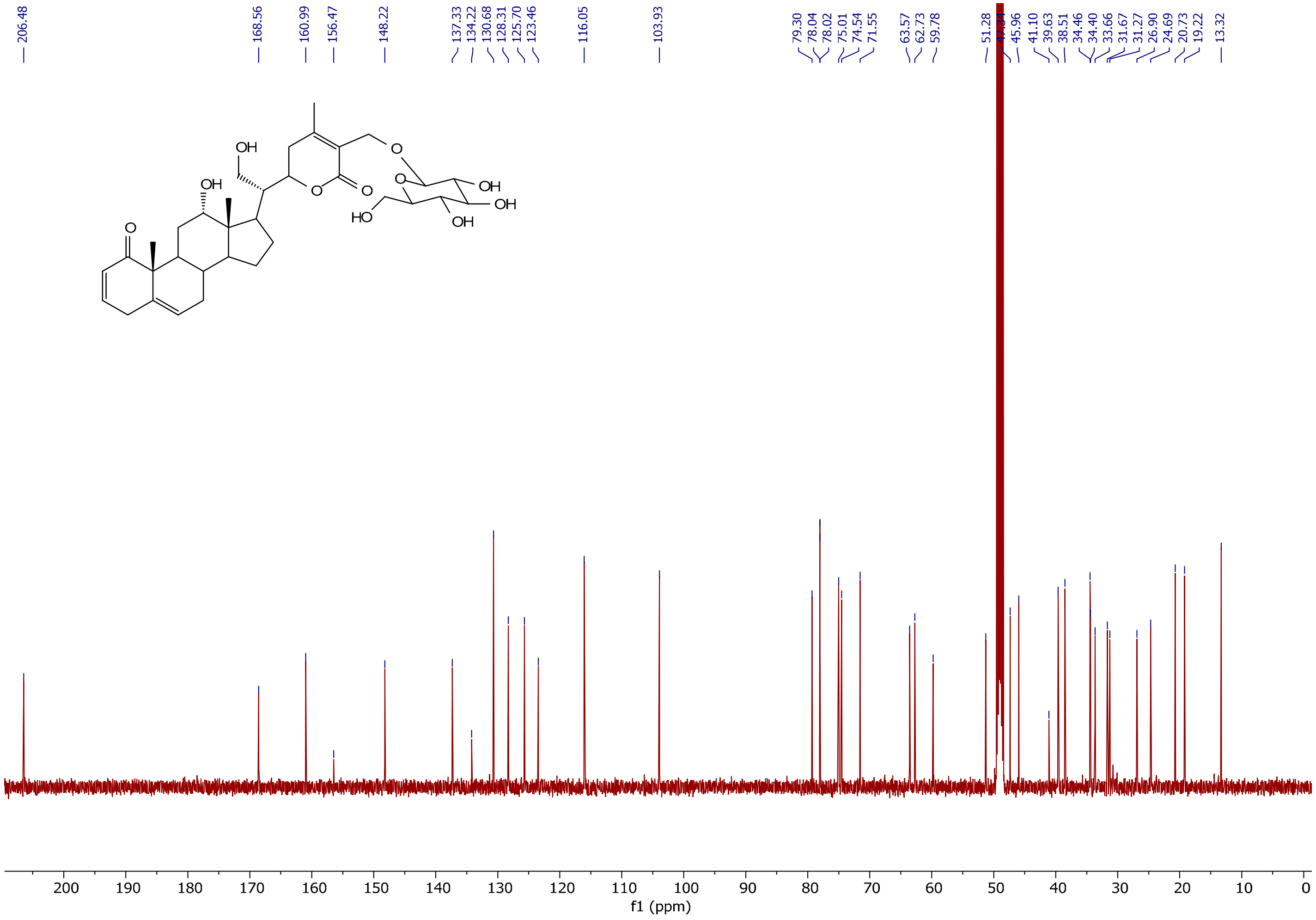

Fig. S3. ^{13}C NMR spectrum (CD_3OD, 125 MHz) of 12a-hydroxydaturametelin B (daturamalakoside B)

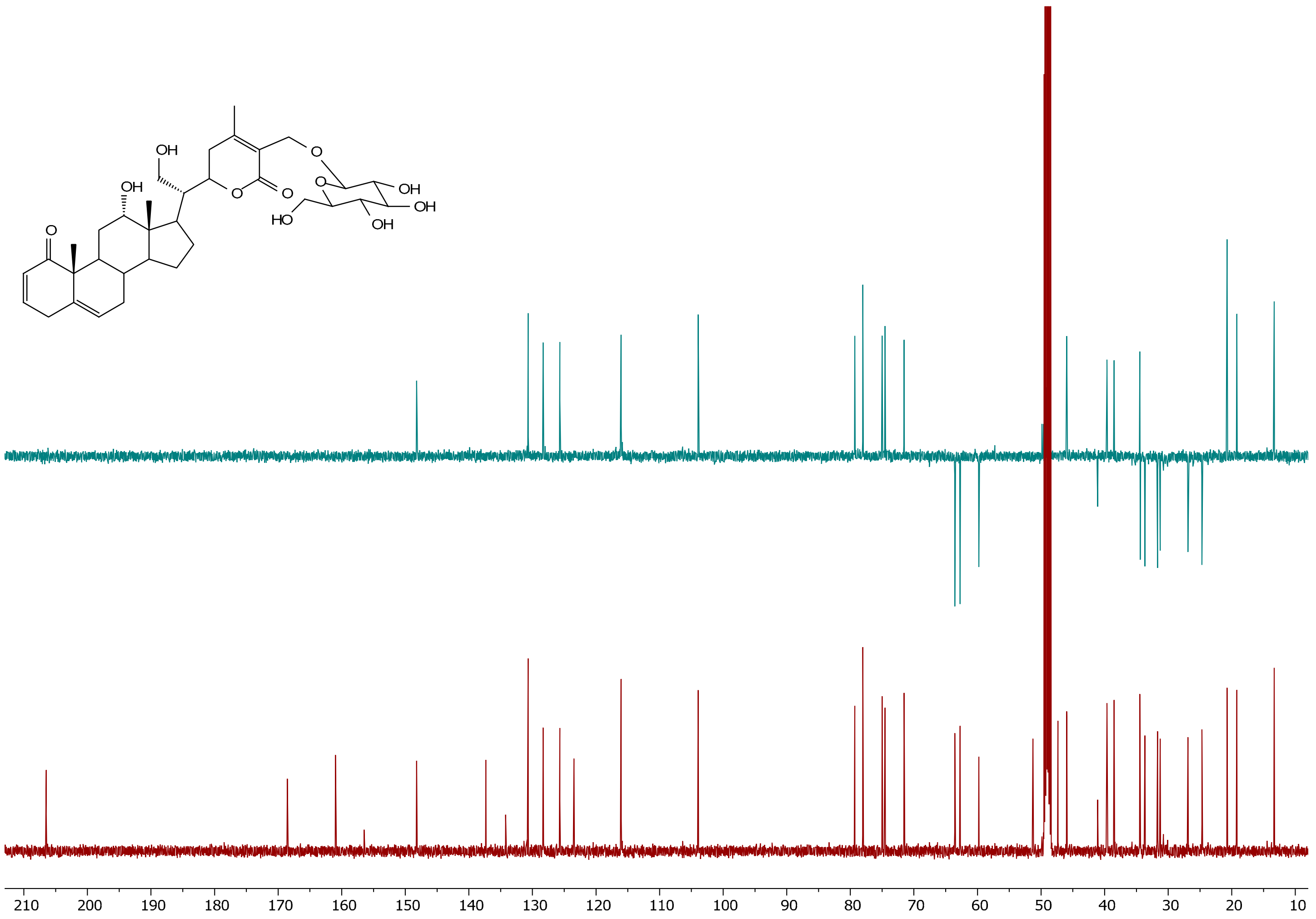

Fig. S4. ^{13}C and DEPT-135 NMR spectra (CD_3OD, 125 MHz) of 12a-hydroxydaturametelin B (daturamalakoside B)

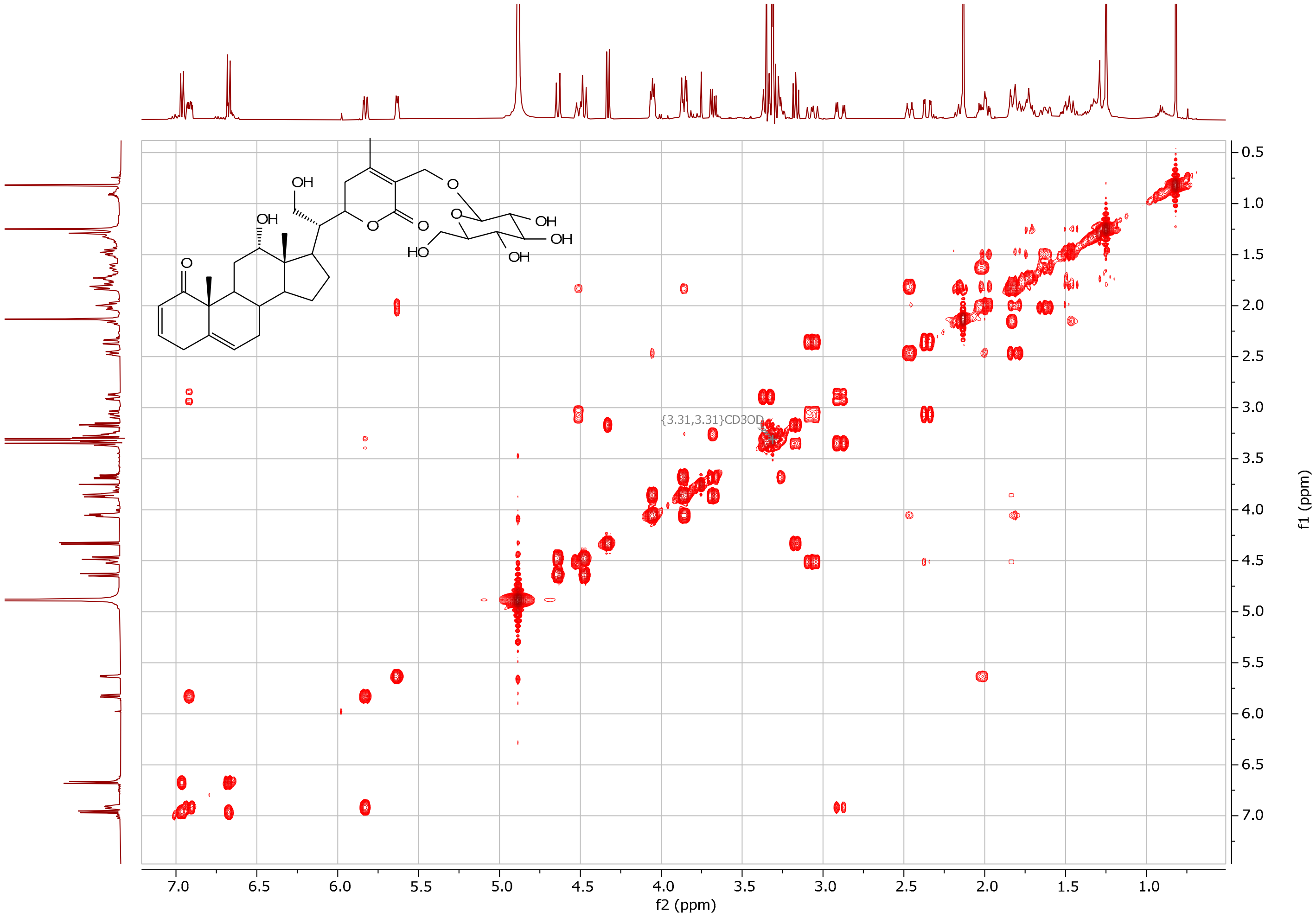

Fig. S5. ^{1}H-^{1}H COSY spectrum (CD_3OD, 500 MHz) of 12a-hydroxydaturametelin B (daturamalakoside B)

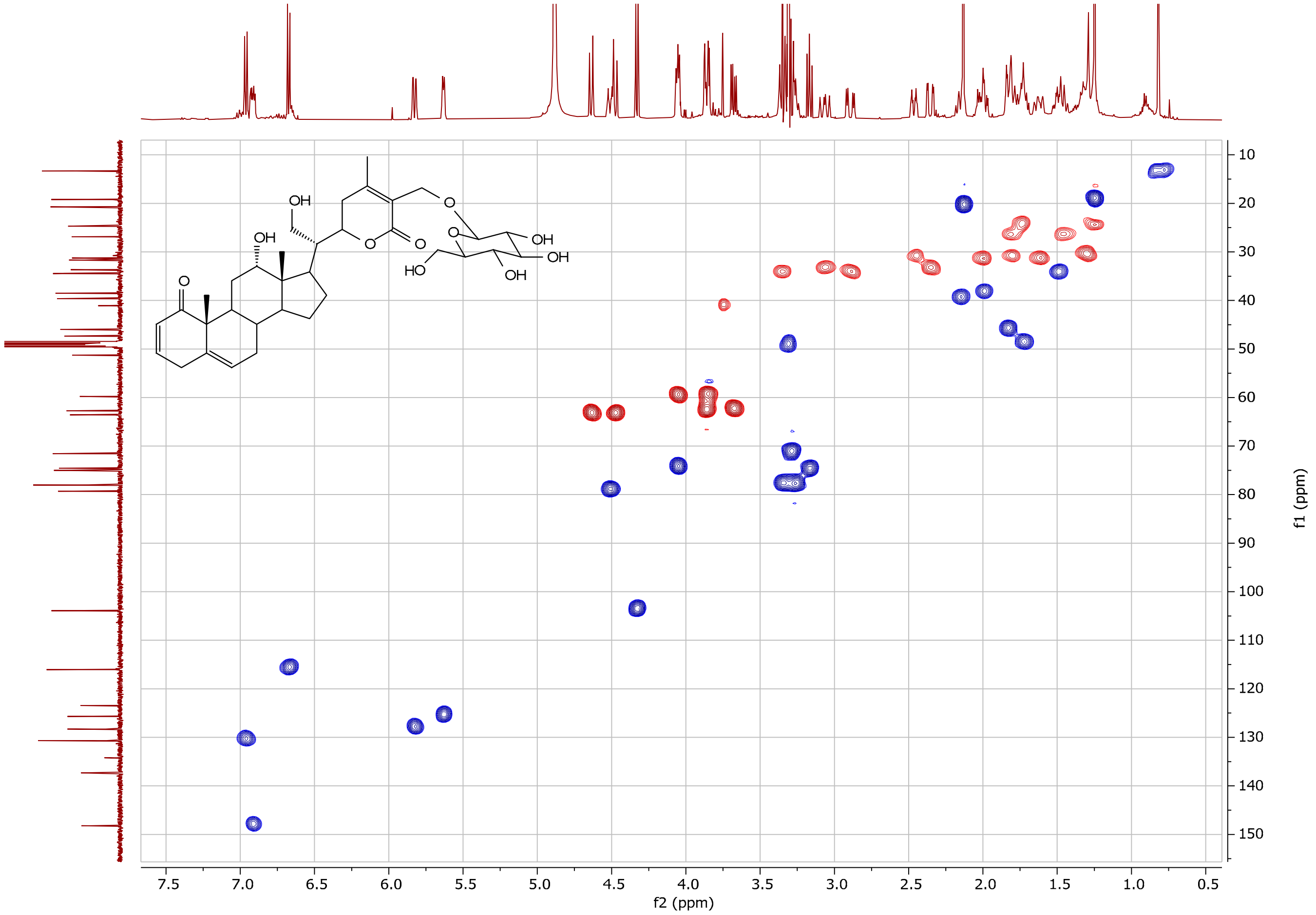

Fig. S6. Edited-HSQC spectrum (CD3OD, 500 MHz) of 12a-hydroxydaturametelin B (daturamalakoside B)

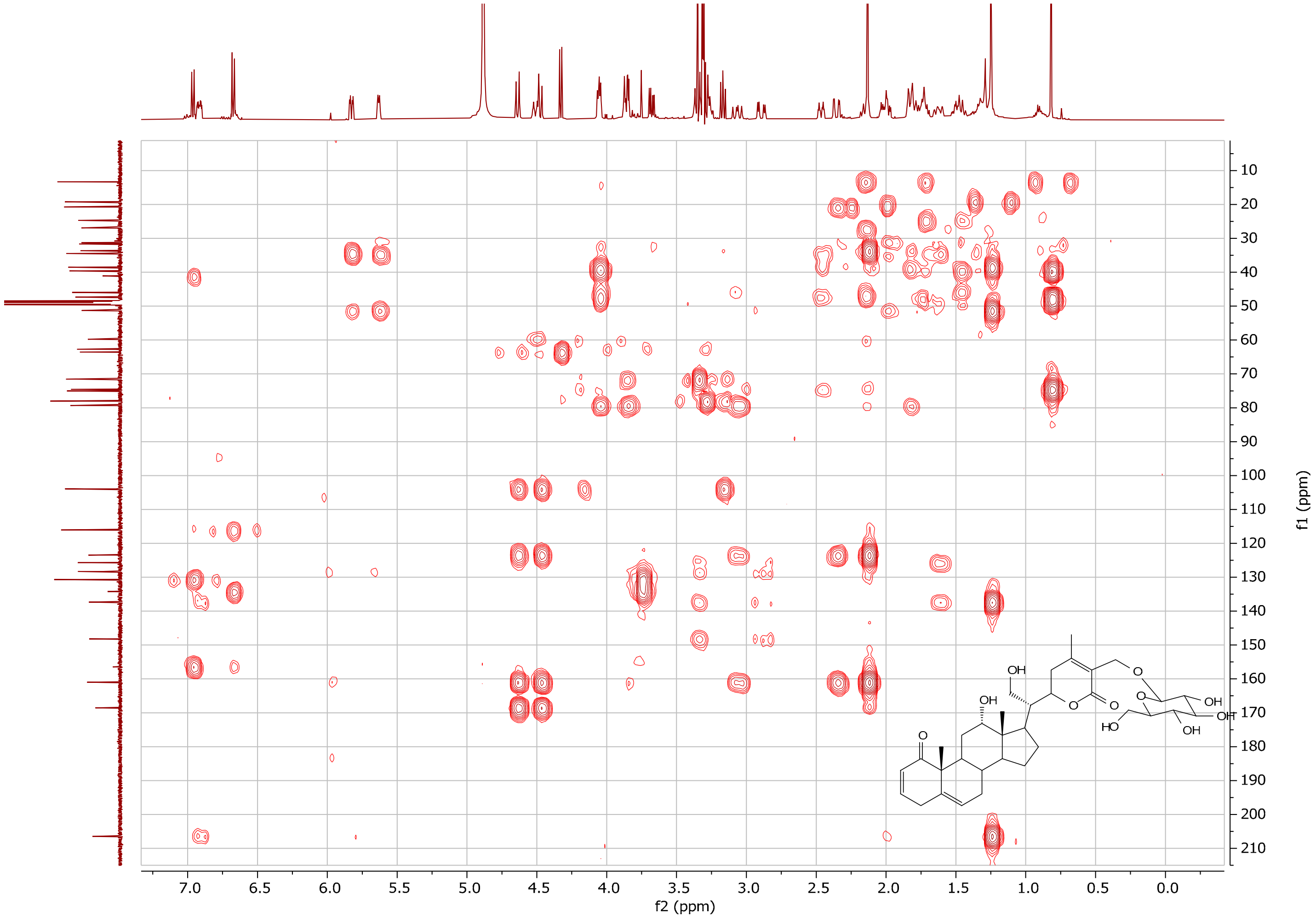

Fig. S7. HMBC spectrum (CD3OD, 500 MHz) of 12a-hydroxydaturametelin B (daturamalakoside B)

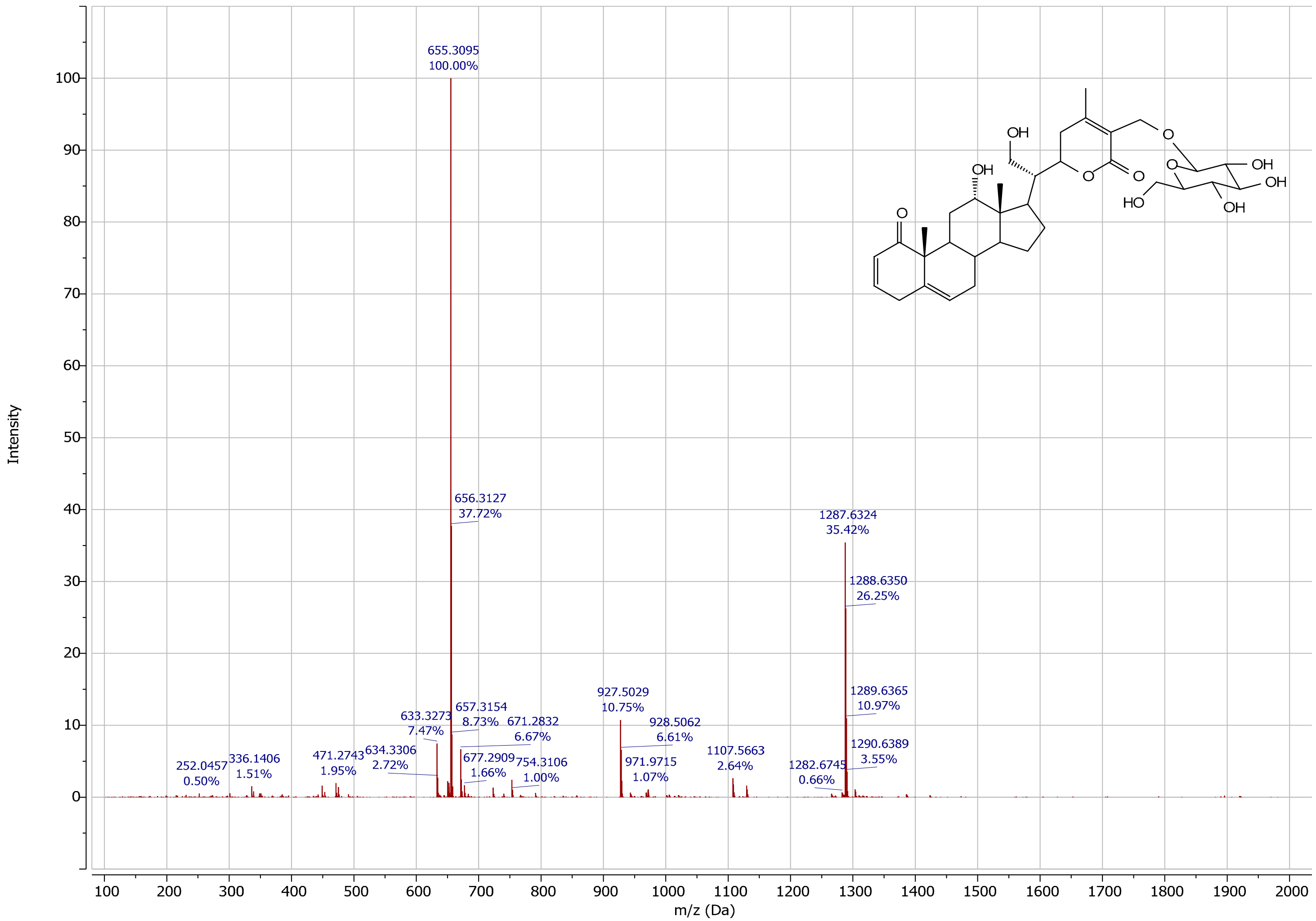

Fig. S8. ESI (+)-HRMS spectrum of 12a-hydroxydaturametelin B (daturamalakoside B)

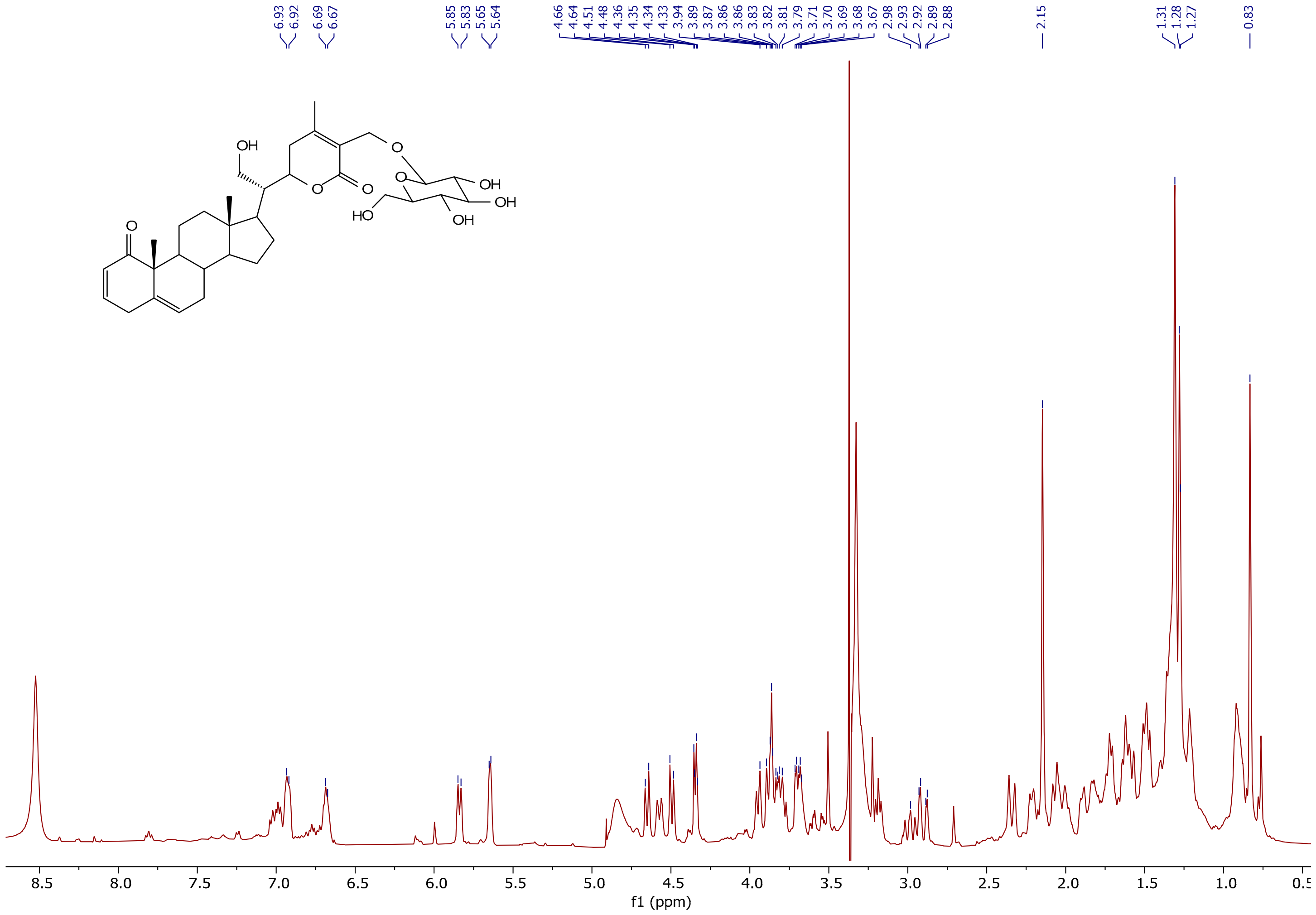

Fig. S9. ^{1}H NMR spectrum (CD_3OD, 500 MHz) of daturametelin B

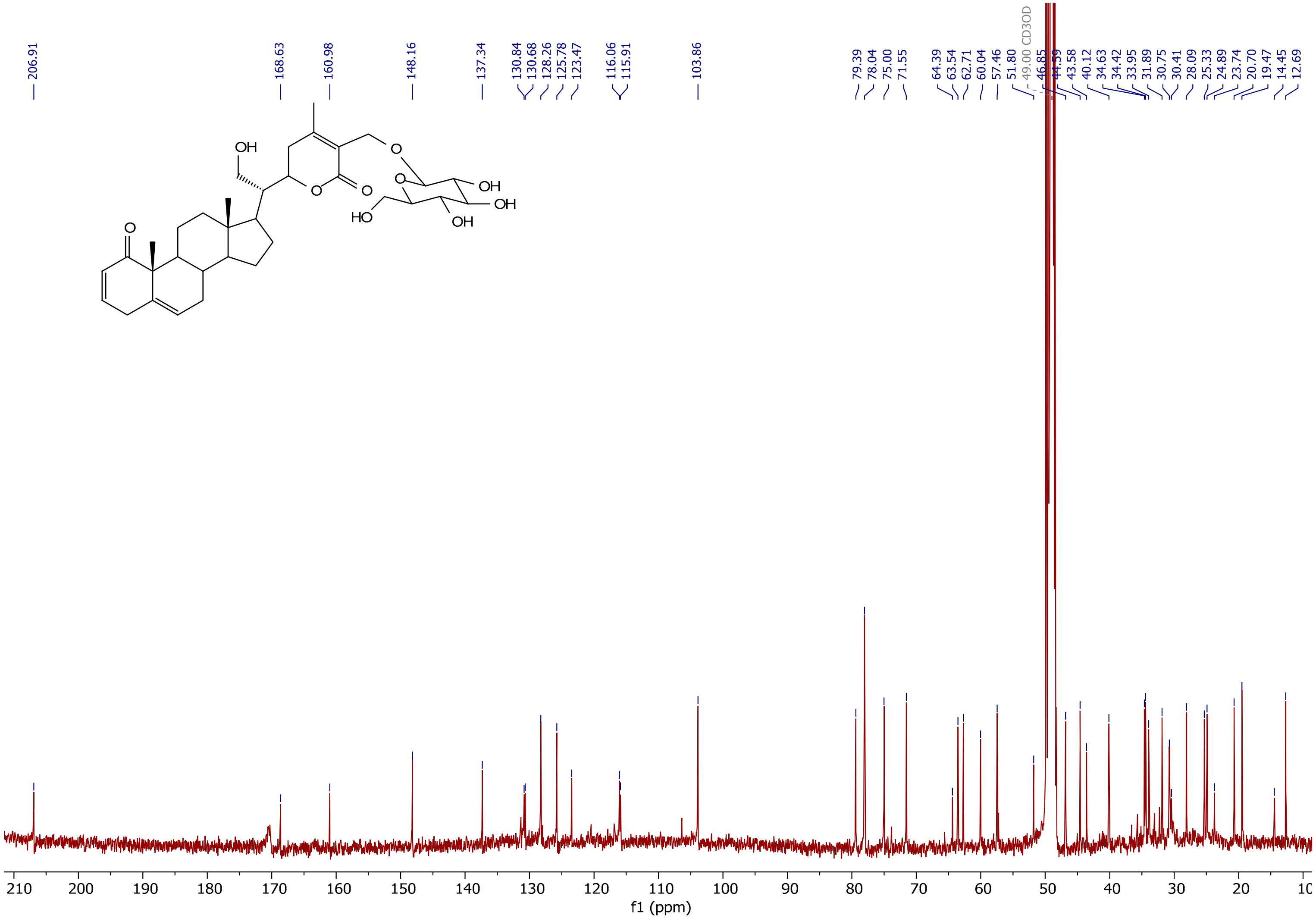

Fig. S10. ^{13}C NMR spectrum (CD_3OD, 125 MHz) of daturametelin B

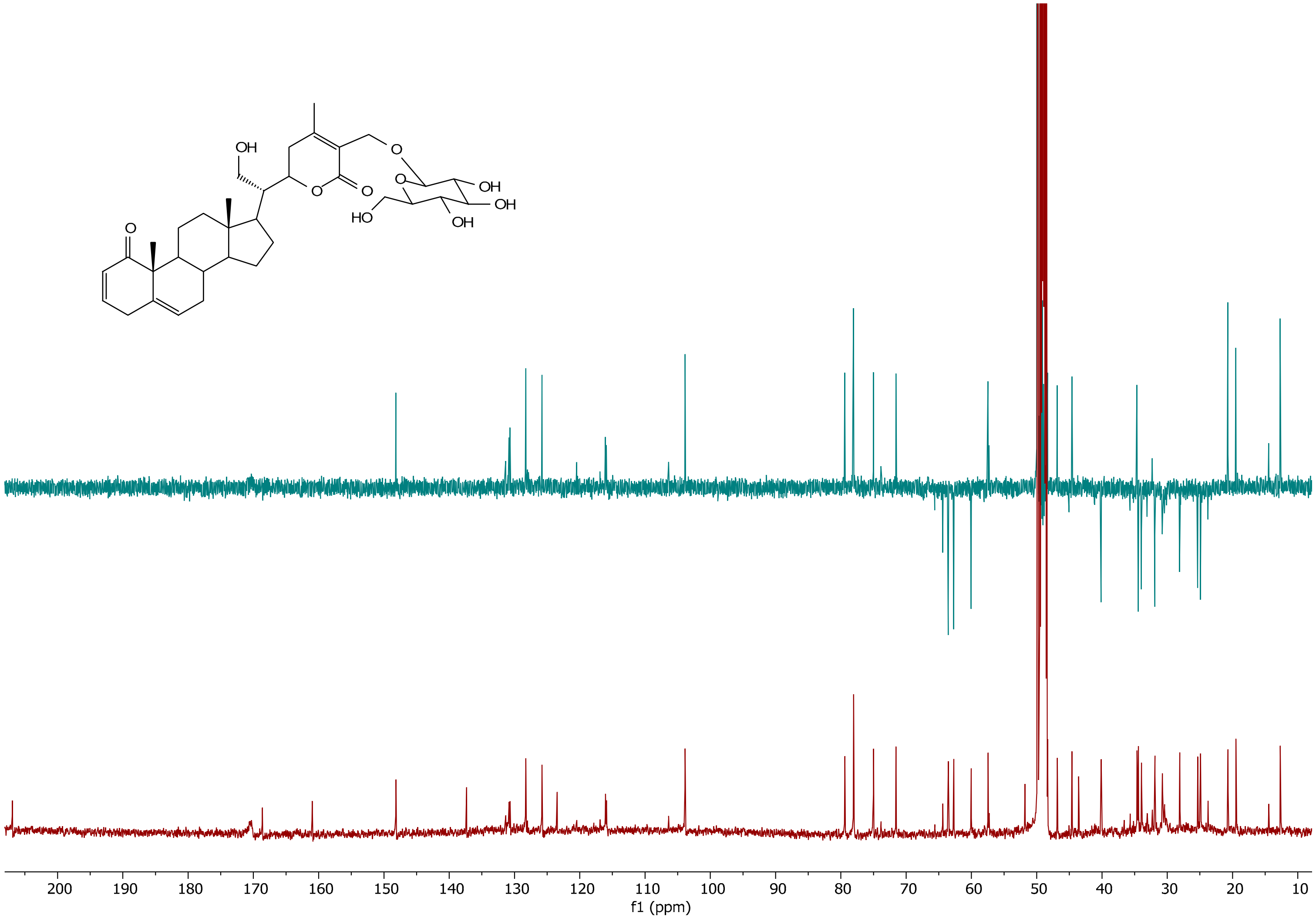

Fig. S11. ^{13}C and DEPT-135 NMR spectra (CD_3OD, 125 MHz) of daturametelin B (P3)

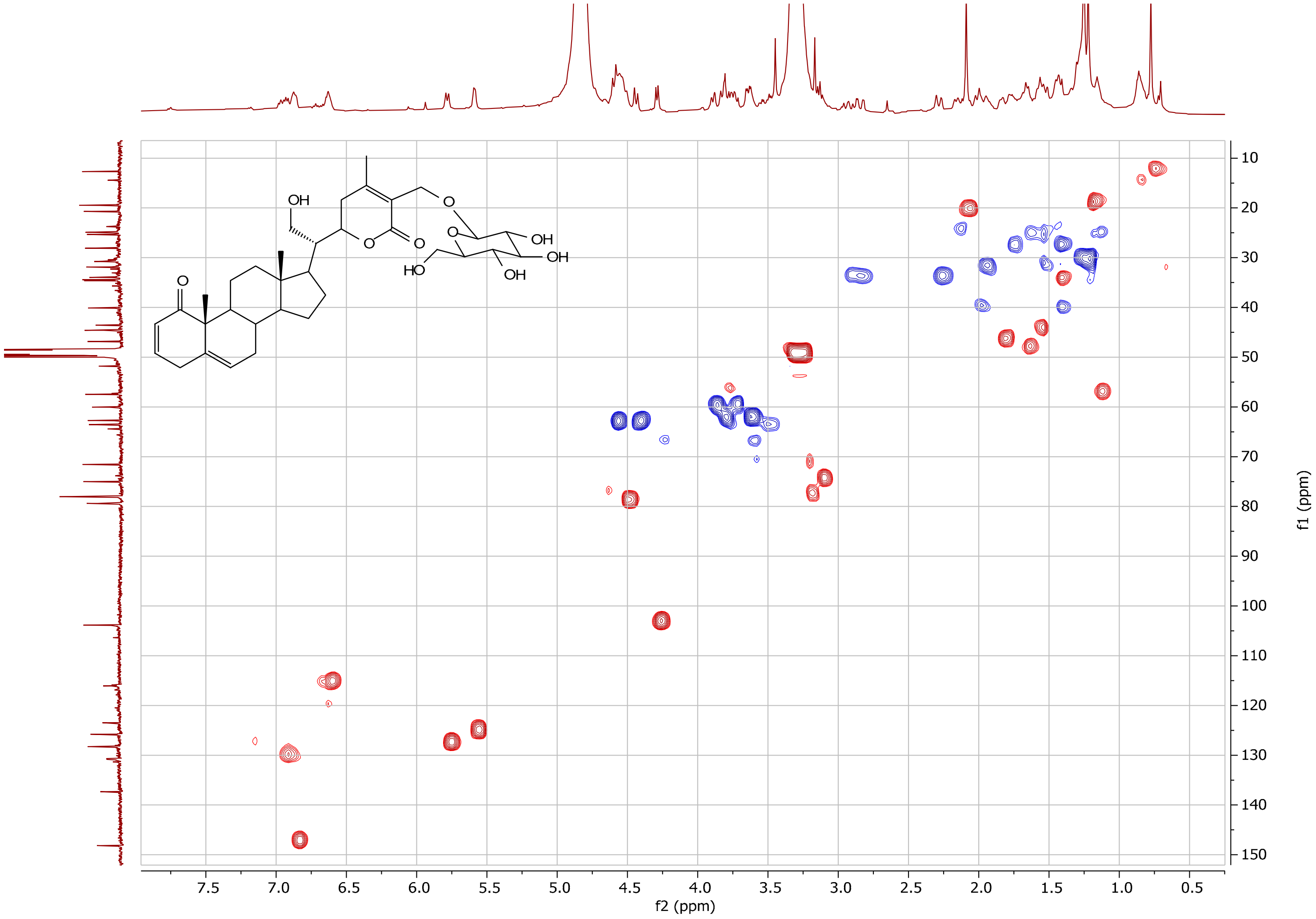

Fig. S12. Edited-HSQC spectrum (CD3OD, 500 MHz) of daturametelin B

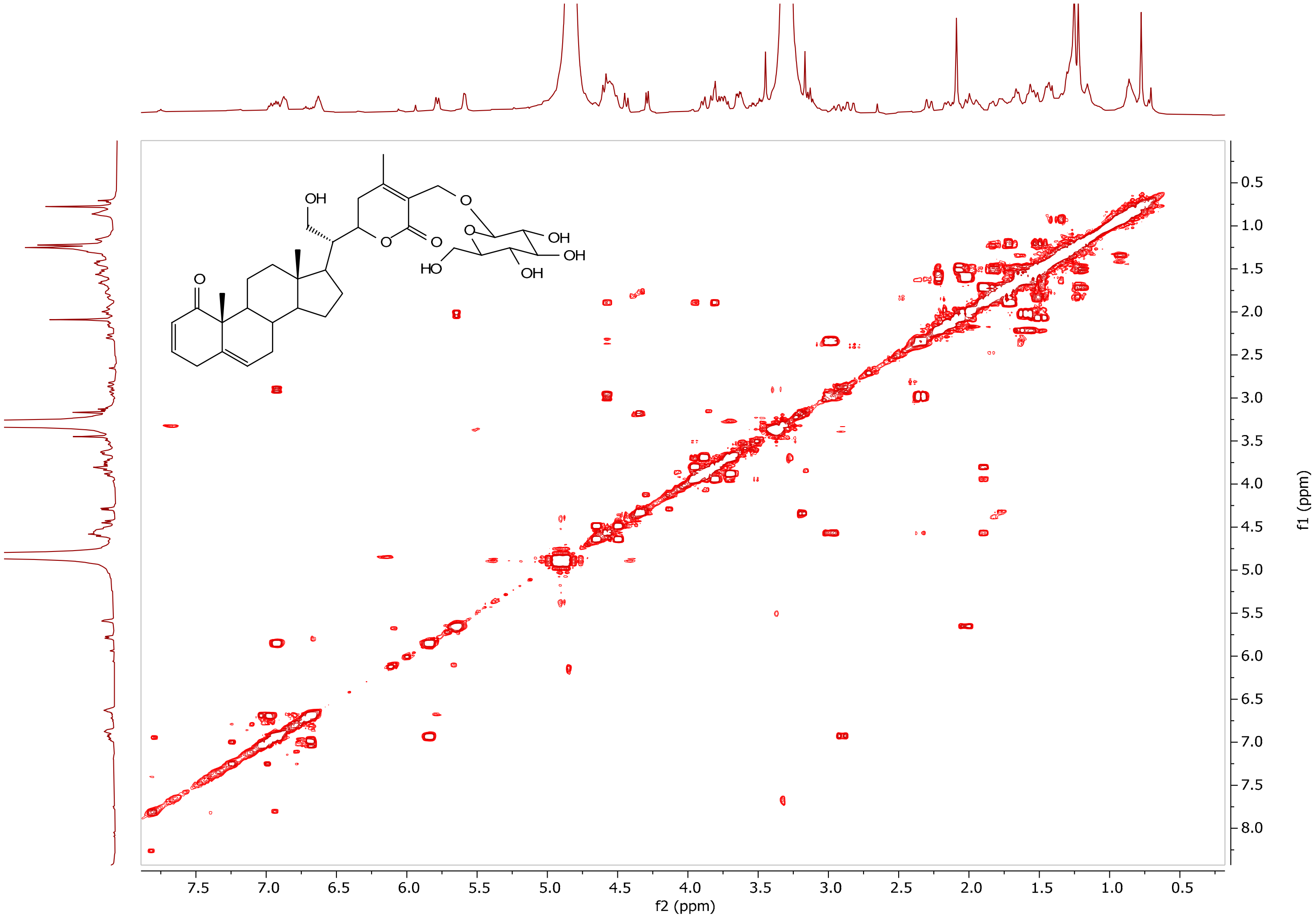

Fig. S13. ^{1}H-^{1}H COSY spectrum (CD_3OD, 500 MHz) of daturametelin B

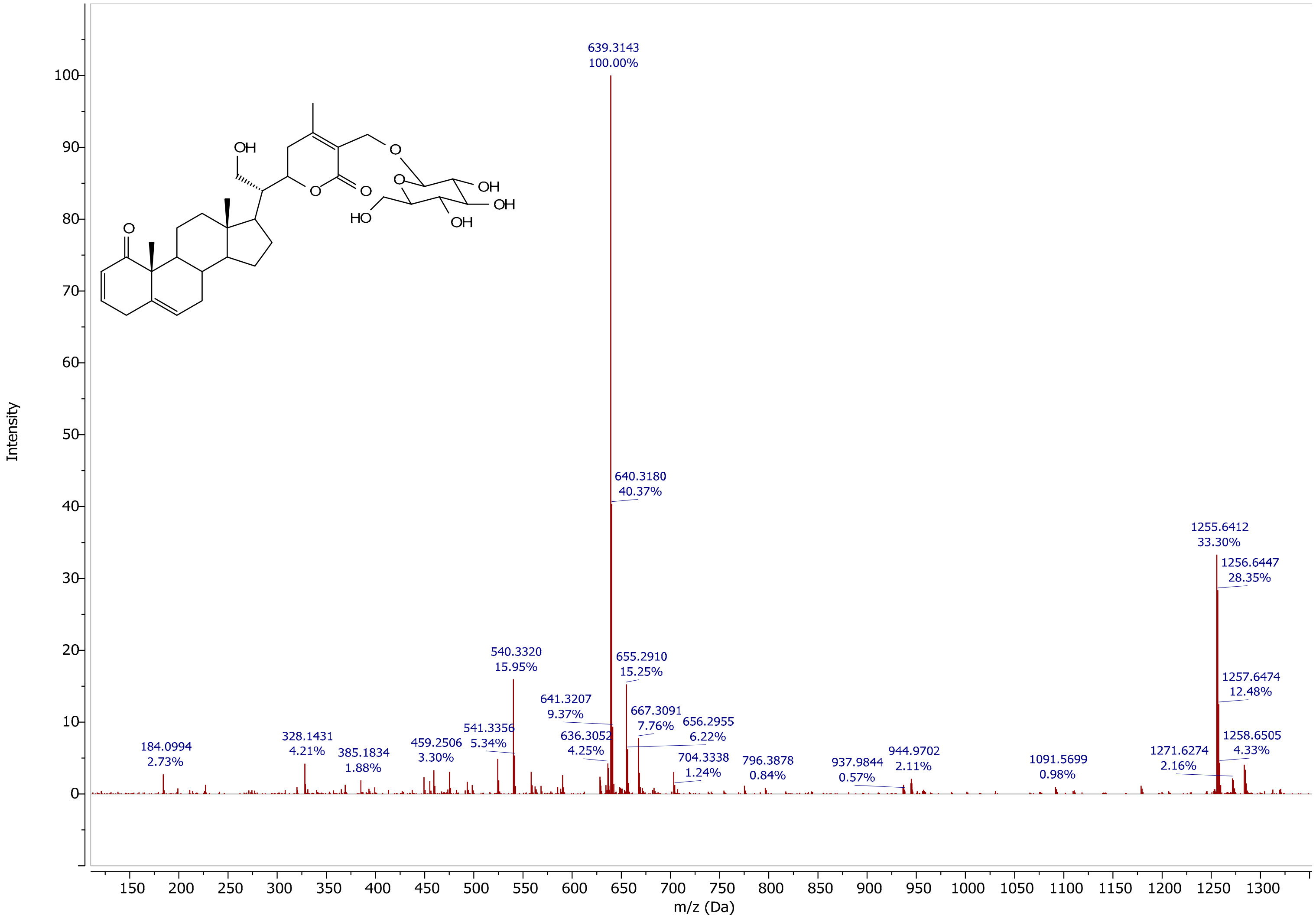

Fig. S14. ESI (+)-HRMS spectrum of daturametelin B

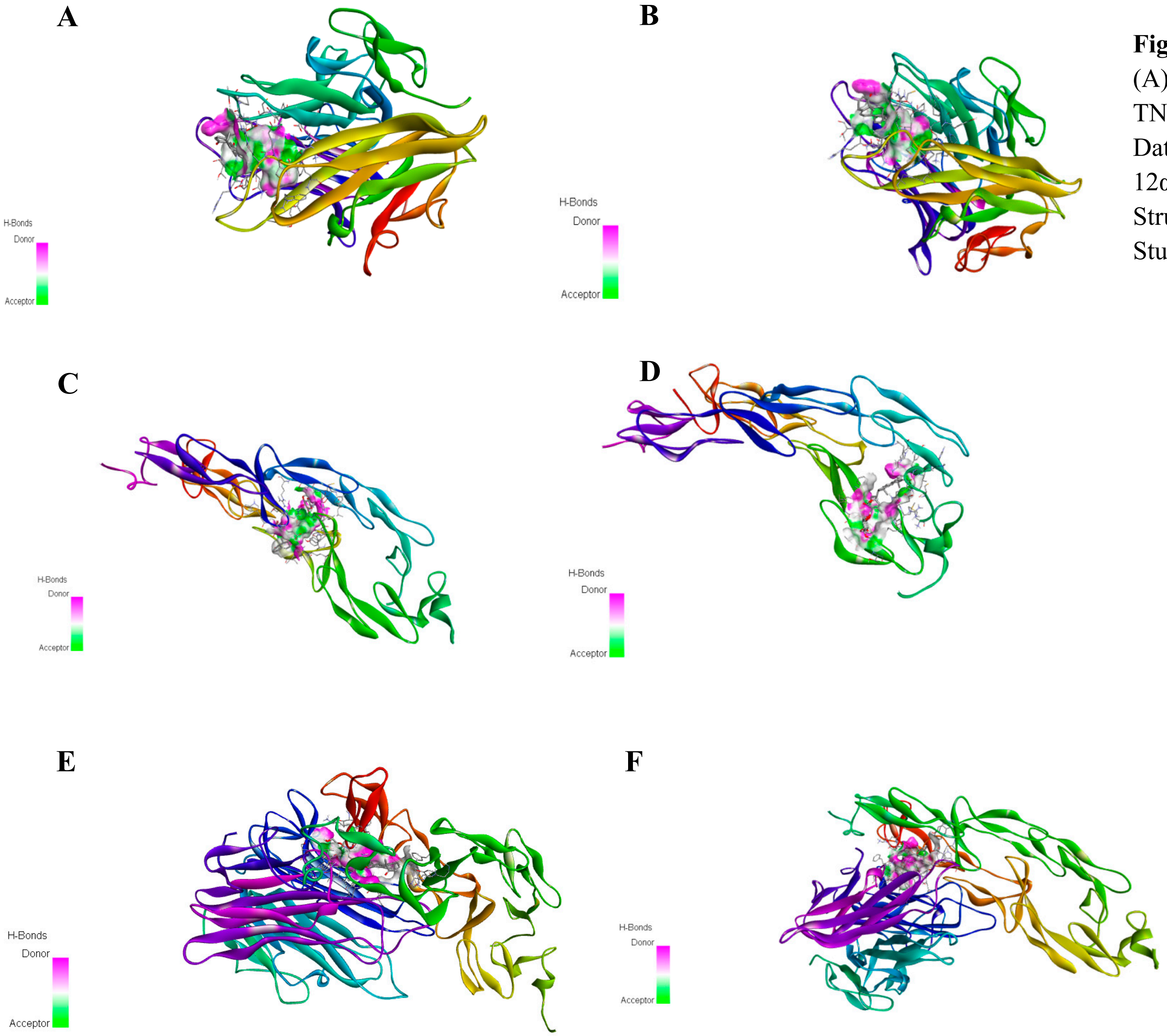

Fig. S15 3D illustration of TNF-α binding poses with (A) 12α-hydroxydaturametelin B, (B) Daturametelin B, TNFR1 with (C) 12α-hydroxydaturametelin B, (D) Daturametelin B and TNF-α- TNFR1complex with (E) 12α-hydroxydaturametelin B, (F) Daturametelin B. Structural graphics were generated using Discovery Studio.2021.

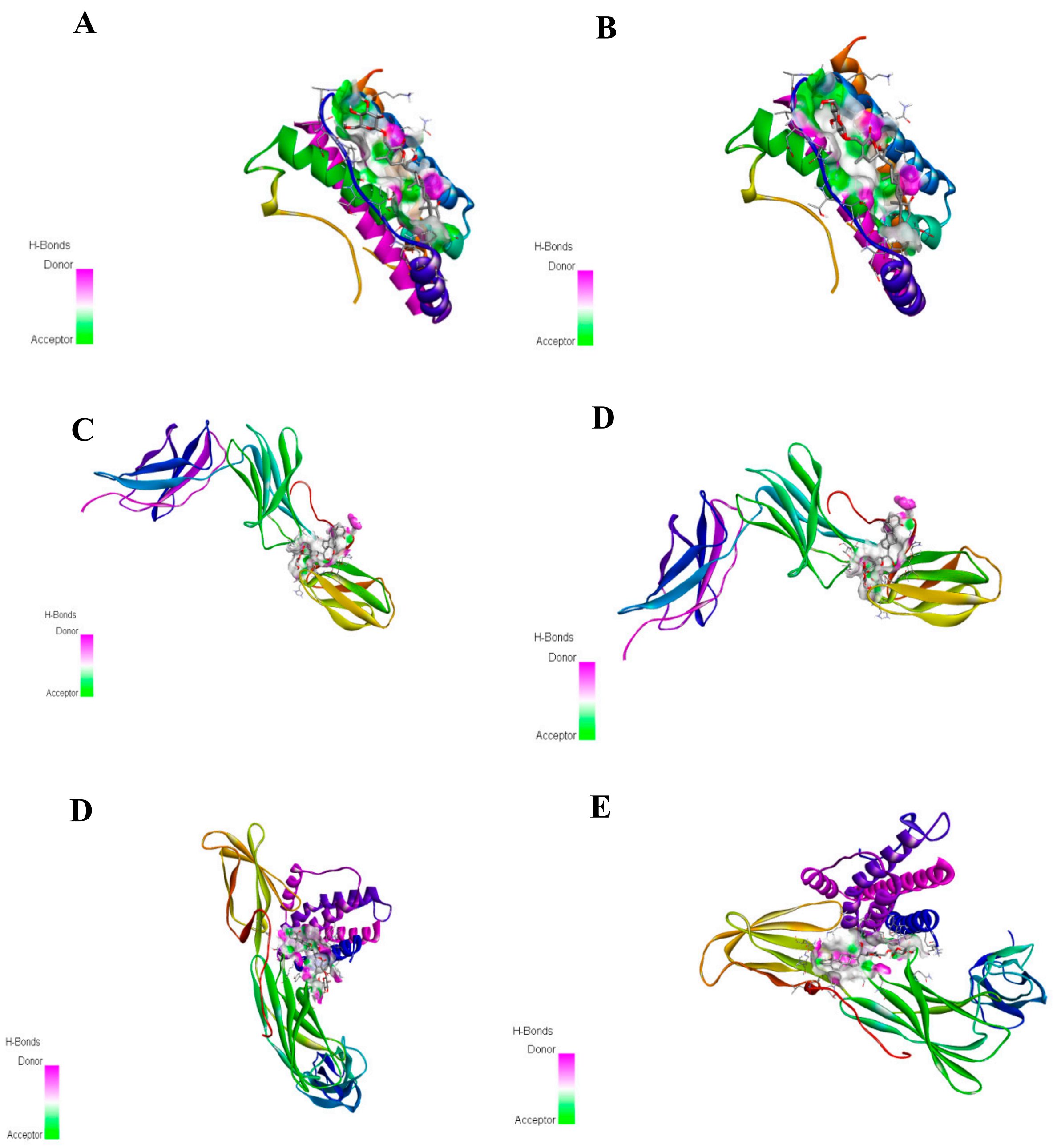

Fig. S16 3D illustration of IL-6 binding poses with (A) 12α-hydroxydaturametelin B, (B) Daturametelin B, IL-6R with (C) 12α-hydroxydaturametelin B, (D) Daturametelin B and IL-6- IL-6R complex with (E) 12α-hydroxydaturametelin B, (F) Daturametelin B. Structural graphics were generated using Discovery Studio.2021.

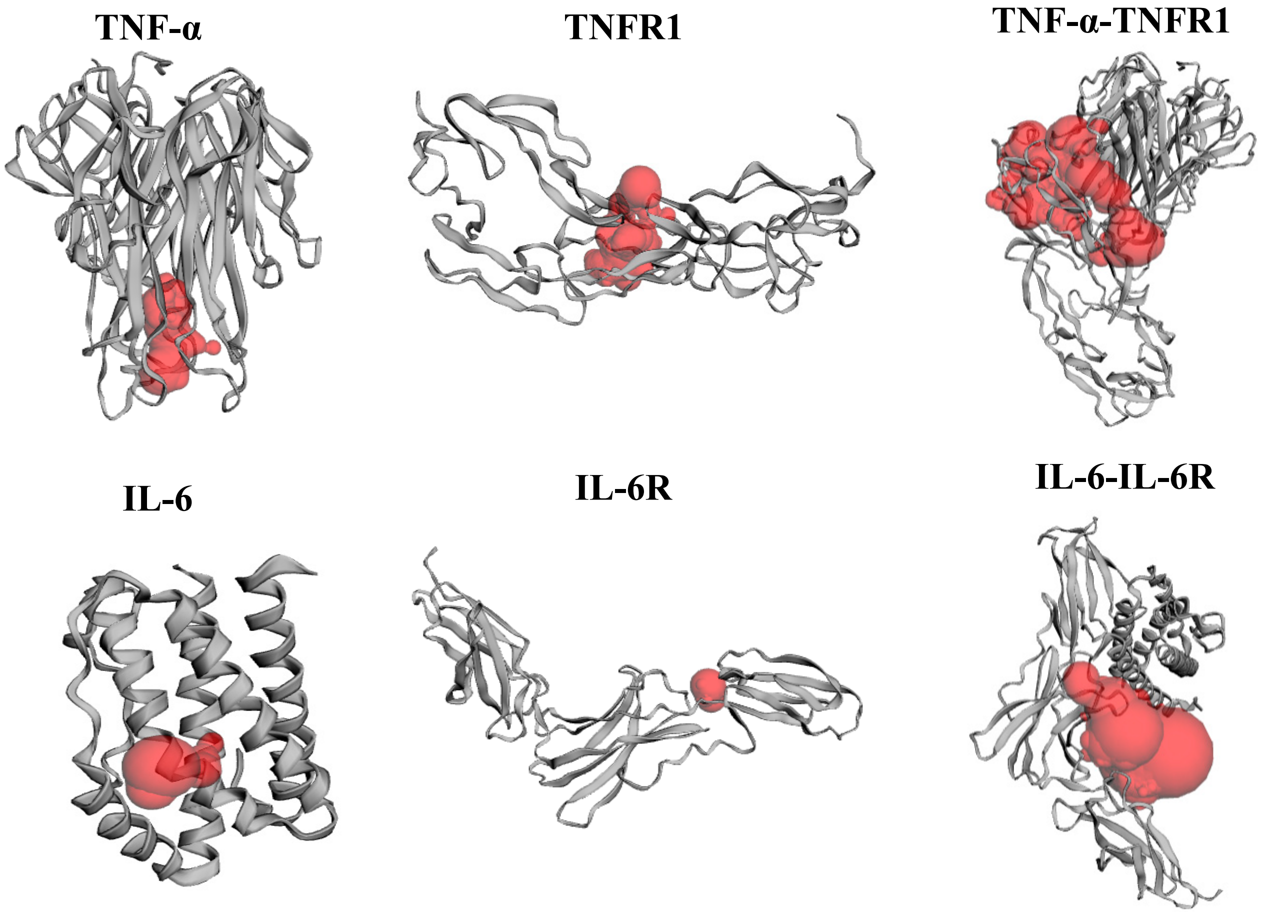

Fig. S17 The active sites of TNF-α, TNFR1, TNF-α–TNFR1 complex, Human IL-6, extra-cellular domains of Human Interleukin-6 Receptor alpha chain (IL-6R) and the IL-6 – IL-6R complex.

Table S1. ^{1}H and ^{13}C NMR Spectral Data of 12α-hydroxydaturametelin B (daturamalakoside B) and daturametelin B

	12α-hydroxydaturametelin B (daturamalakoside B)		*Daturametelin B)*	
	**δ_C, mult**	**δ_H, mult (*J* in Hz)**	**δ_C, mult**	**δ_H, mult (*J* in Hz)**
**1**	206.48, C	-	206.91, C	-
**2**	128.30, CH	5.83 dd (10.1, 1.8)	128.26, CH	5.82 d (9.9)
**3**	148.22 CH	6.90 m	148.16, CH	6.92 bs
**4**	34.40, CH_2	2.89 dd (21.0, 5.2)/3.34 m	34.42, CH_2	-
**5**	137.34, C	-	137.34, C	-
**6**	125.68, CH	5.63 bs	125.78, CH	5.64 bd
**7**	31.69, CH_2	2.01 m/1.63 m	30.75, CH_2	
**8**	34.50, CH	1.48 m	34.63, CH	
**9**	38.48, CH	1.99 m	44.54, CH	
**10**	51.09, C	-	51.80, C	
**11**	31.25, CH_2	2.47 dt (14.4, 3.6)/1.79 m	23.74, CH_2	
**12**	74.51, CH	4.045 bs	40.09, CH_2	
**13**	47.28, C	-	43.58, C	
**14**	48.83, CH	1.72 m	32.27, CH	
**15**	24.34, CH_2	1.74 m/ 1.26 m	24.89, CH_2	
**16**	26.89, CH_2	1.81 m/ 1.46 m	25.33, CH_2	
**17**	39.61, CH	2.14 m	46.85, CH	
**18**	13.32, CH_3	0.81 s	12.69, CH_3	0.83 s
**19**	19.22, CH_3	1.23 s	19.47, CH_3	1.27 s
**20**	45.90, CH	1.82 m	44.59, CH	
**21**	59.78, CH_2	4.05 d (11.0)/3.86 m	60.04, CH_2	4.52 m/3.90 d (11.5)
**22**	79.30, CH	4.50 m	79.39, CH	
**23**	33.64, CH_2	3.07 dd (18.5, 13.1)/2.35 dd(18.5, 3.2)	33.95, CH_2	
**24**	160.99, C	-	160.98, C	
**25**	123.45, C	-	123.47, C	
**26**	168.53, C	-	168.63, C	
**27**	63.57, CH_2	4.64 d (11.2)/4.48 d (11.2)	63.54, CH_2	4.65 d (11.5)/4.45 d(11.5)
**28**	20.73, CH_3	2.13 s	20.70, CH_3	2.15 s
**1'**	103.93, CH	4.33 d (7.7)	103.86, CH	4.32 d (7.7)
**2'**	75.00, CH	3.17 t (8.5)	75.00, CH	3.16 d (8.4)
**3'**	78.04, CH	3.33 m	78.04, CH	3.23 m
**4'**	71.55, CH	3.28 m	71.55, CH	3.25 m
**5'**	78.08, CH	3.25 m	78.04, CH	3.31 m
**6'**	62.73, CH_2	3.87 m/ 3.68 dd (11.8, 5.3)	62 .71, CH_2	3.84 m / 3.67 dd (12.4, 5.5)

Table S2 12α-hydroxydaturametelin B (daturamalakoside B) and daturametelin B characteristics and structures obtained from PubChem Sketcher V2.4.

Sample Name	12α-hydroxydaturametelin B (daturamalakoside B)	Daturametelin B
**molecular formula**	$C_{34}H_{48}O_{11}$	$C_{34}H_{48}O_{10}$
***m/z* exp**	655.3095 $[M+Na]^+$	639.3143 $[M+Na]^+$
***m/z* calcd**	655.3088 $[M+Na]^+$	639.3081 $[M+Na]^+$
**[ɒ]D[a]**	-	+1.40°
**Isomiric SMILES[b]**	CC1=C(C(=O)OC(C1)[C@@H](CO)[C@H]2CC[C@@H]3[C@@]2([C@@H](C[C@H]4[C@H]3CC=C5[C@@]4(C(=O)C=CC5)C)O)C)CO[C@H]6[C@@H]([C@H]([C@@H]([C@H](O6)CO)O)O)O	CC1=C(C(=O)OC(C1)[C@@H](CO)[C@H]2CC[C@@H]3[C@@]2(CC[C@H]4[C@H]3CC=C5[C@@]4(C(=O)C=CC5)C)C)CO[C@H]6[C@@H]([C@H]([C@@H]([C@H](O6)CO)O)O)O

[a]KazushiShingu et al., (1987)

[b]PubChem Sketcher V2.4

Table S3 Grid box parameters selected for the target proteins

Protein	PDB ID	Resolution (Å)	Grid Box Center Coordinates			Grid Box Size
			x	y	z	
**TNF α**	1TNF	2.60	10.124	57.015	32.343	40 x 62 x 72
**TNF receptor 1**	1EXT	1.85	-0,205	32.613	-4,696	64 X 56 X 58
**IL-6**	1ALU	1.90	7.534	-22.268	15.698	32 x 32 x 28
**IL-6 Receptor alpha chain**	1N26	2.35	38.819	66.779	63.68	40 x 40 x 28
		**PyDock**				
**Protein-Protein complex**	JOBID	Electrostatics	x	y	z	**Grid Box Size**
**TNFα-TNFR1complex**	J_6374AD30186DE	-18.667	-8.781	44.205	-17.314	58 X 28 X 54
**IL-6-IL-6R complex**	J_637781CCAE854	-18.241	29.572	65.813	93.314	48 x 36 x 66
